# Antagonistic interactions between phage and host factors control arbitrium lysis–lysogeny decision

**DOI:** 10.1038/s41564-023-01550-4

**Published:** 2024-01-04

**Authors:** Sara Zamora-Caballero, Cora Chmielowska, Nuria Quiles-Puchalt, Aisling Brady, Francisca Gallego del Sol, Javier Mancheño-Bonillo, Alonso Felipe-Ruíz, Wilfried J. J. Meijer, José R. Penadés, Alberto Marina

**Affiliations:** 1 Instituto de Biomedicina de Valencia (IBV)-CSIC and CIBER de Enfermedades Raras (CIBERER)-ISCIII, Valencia, Spain; 2 Centre for Bacterial Resistance Biology, Imperial College London, London, UK; 3 Department of Biomedical Sciences, Faculty of Health Sciences, Universidad CEU Cardenal Herrera, CEU Universities, Alfara del Patriarca, Spain; 4 Institute of Infection, Immunity and Inflammation, University of Glasgow, Glasgow, UK; 5 Centro de Biología Molecular “Severo Ochoa” (CSIC-UAM), Universidad Autónoma, Cantoblanco, Madrid, Spain

**Keywords:** Phage biology, X-ray crystallography, Microbial ecology, Molecular biophysics, Bacterial transformation

## Abstract

Phages can use a small-molecule communication arbitrium system to coordinate lysis–lysogeny decisions, but the underlying mechanism remains unknown. Here we determined that the arbitrium system in *Bacillus subtilis* phage phi3T modulates the bacterial toxin–antitoxin system MazE–MazF to regulate the phage life cycle. We show that phi3T expresses AimX and YosL, which bind to and inactivate MazF. AimX also inhibits the function of phi3T_93, a protein that promotes lysogeny by binding to MazE and releasing MazF. Overall, these mutually exclusive interactions promote the lytic cycle of the phage. After several rounds of infection, the phage-encoded AimP peptide accumulates intracellularly and inactivates the phage antiterminator AimR, a process that eliminates *aim*X expression from the *aim*P promoter. Therefore, when AimP increases, MazF activity promotes reversion back to lysogeny, since AimX is absent. Altogether, our study reveals the evolutionary strategy used by arbitrium to control lysis–lysogeny by domesticating and fine-tuning a phage-defence mechanism.

## Abstract (web summary)

The phage-encoded arbitrium communication system controls the activity of the host bacterial toxin–antitoxin system MazE–MazF to regulate lysis–lysogeny decision.

## Main

Bacterial viruses (phages) parasitize their host’s machinery to replicate and produce progeny, a process resulting in lysis of the cell. Temperate phages possess an alternative stage to the lytic cycle, termed lysogeny, where the viral DNA integrates into the host chromosome and becomes dormant. The genetic determinants that govern the lysis–lysogeny decision are well understood in the *Escherichia coli* model temperate phage Lambda^1^, but less is known for Gram-positive infecting phages^2^. Recently, it was discovered that some *Bacillus*-infecting phages encode a peptide-based communication system, named arbitrium, which allows the virus to ‘talk’ to their kin and coordinate the lysis–lysogeny decision by incorporating the population-sensing factor into this process^3^. Although originally described in phages of the SPbeta group (phi3T and SPβ), the arbitrium system is widespread in Bacillota and can also be found in conjugative elements and plasmids^4^.

In the phi3T phage, the arbitrium system consists of three main components, all encoded in the phage genome^3^. AimP, the small signalling peptide, is secreted into the medium after phage infection and is internalized by surrounding bacteria. The sensor, AimR, is a transcription factor that in the absence of AimP promotes the expression of *aim*X, which exerts a negative regulatory effect on lysogeny by an unknown mechanism. When the host:phage ratio is high during initial stages of the phage infection, AimP peptide concentration is low, *aim*X is expressed and the lytic cycle is favoured^3^. Accumulation of the peptide allows the phage to preferentially switch to lysogeny when the susceptible bacterial population is reduced, which allows safe persistence of both the integrated prophage and the bacterial host^3^. AimP binds to AimR^5^, inactivating its function and therefore inhibiting the expression of *aim*X, which results in promoting lysogeny. However, the molecular details of this mechanism remain elusive.

We initiated this study trying to understand how *aim*X controls the lysis–lysogeny decision in phage phi3T. To our surprise, our results not only redefine roles for *aim*X and AimR, as well as propose an additional function for *aim*P, but also bring forward new phage- and host-encoded players. The mutually exclusive interactions between these players are essential for the functionality of the arbitrium system and provide important insights into how the arbitrium system controls lysis–lysogeny decisions in phage communication.

## Results

### *aimX* expression is regulated by a transcriptional terminator

**Extended Data Fig. 1:**
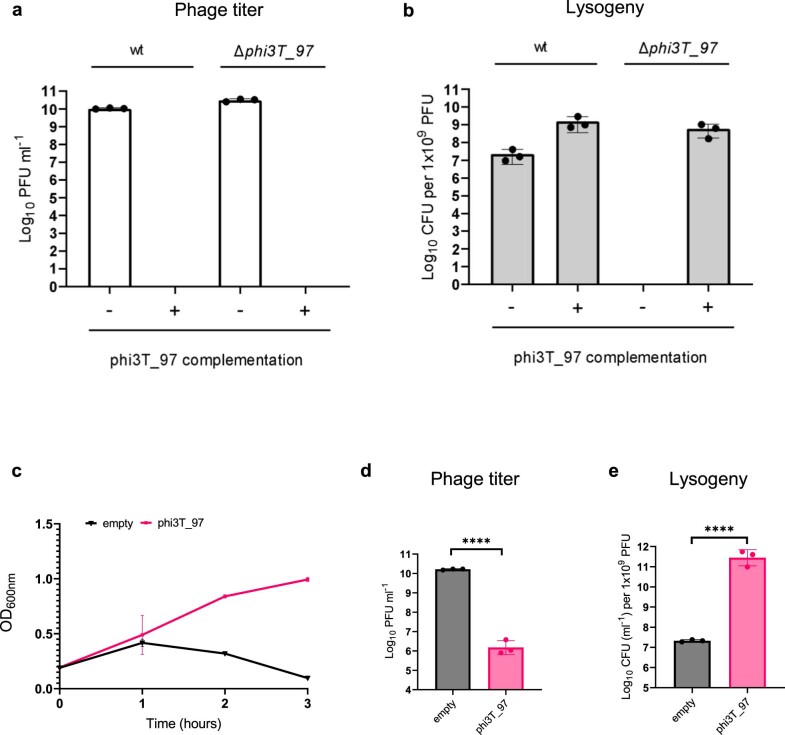
Characterisation of phi3T master repressor. **(a-b)** Lysates containing phages phi3T wt or phi3T Δ*phi3T_97* were used to infect strains 168 Δ6 or 168 Δ6 *amy*E::P_Spank_-phi3T_97, which overexpresses phi3T_97. The number of resulting plaques and lysogens were then quantified. The geometric mean and SD are presented (n = 3). **(c)** *B. subtilis* strains either devoid of (black) or expressing phi3T_97 (pink) were infected with phage phi3T wt at an MOI of 1:10 (phage:bacteria). The mean and SD of OD_600_ readings (n = 3) are presented. **(d-e)** After 3 h, the number of resulting plaques and relative number of lysogens were quantified. The geometric mean and SD are presented (n = 3). The results are shown as PFUs ml^-1^ or as CFUs ml^−1^ normalised by PFUs ml^-1^ and represented as the CFU of an average phage titer (1×10^9^ PFU ml^-1^). Values below detection limit (1 ×10^1^ PFUs ml^−1^ or CFUs ml^−1^) are not marked. In panel (d) and (e) a two-tailed t-test was performed to compare mean differences; p values indicated were as follows: ****p < 0.0001. Source data

**Extended Data Fig. 2:**
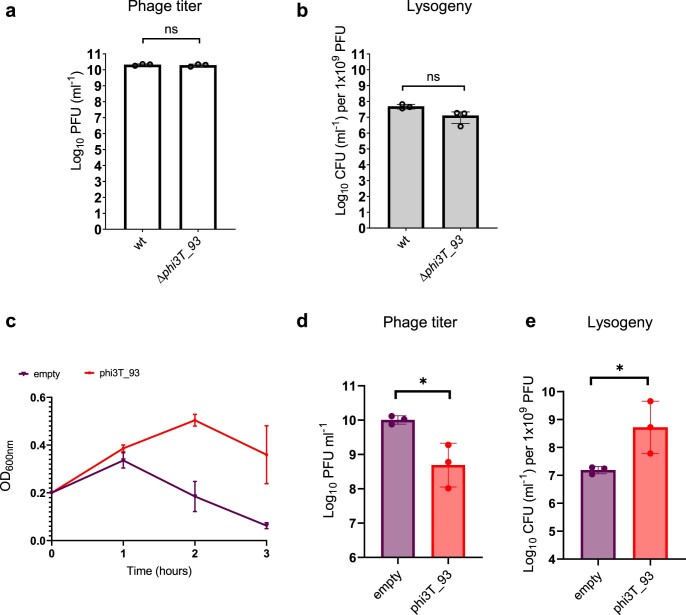
Characterisation of phi3T_93. **(a-b)** 168 Δ6 strains lysogenic for wt phi3T and phi3T mutant Δ*phi3T_93* were MC induced. The number of resulting phages were quantified using 168 Δ6 as recipient strain. The number of resulting plaques (PFUs ml^−1^) and lysogens (CFUs ml^−1^) are presented and geometric mean and SD (n = 3) are marked. A two-tailed t-test of log_10_ -transformed data was performed to compare mean differences. p values indicated are as follows: ns (not significant). **(c)** *B. subtilis* strains either devoid of (purple) or expressing phi3T_93 (pink) were infected with phage phi3T wt at an MOI of 1:10 (phage:bacteria). The mean and SD of OD_600_ readings are presented (n = 3). **(d-e)** After 3 h the number of resulting plaques and relative number of lysogens were quantified. Geometric mean and SD are presented in panels (d) and (e). The results are shown as PFUs ml^-1^ or as CFUs ml^−1^ normalised by PFUs ml^-1^ and represented as the CFU of an average phage titer (1×10^9^ PFU ml^-1^). Two-tailed t-tests were performed to compare mean differences; p values indicated were as follows: *p < 0.05. Results presented in panels (c-e) are a subset of a more extended experiment, presented in full in Fig.5. Source data

Our previous study identified two additional key components of the lysis–lysogeny decision system in phage SPβ, *yop*R and *yop*N, which are part of a six-gene operon (*yop*M–*yop*R), conserved in the SPbeta group of phages. YopR is the master repressor of SPβ, while YopN expression favours lysogenization by an unknown mechanism^6^. This operon in phi3T comprises genes *phi3T_92* to *phi3T_97* (ref. ^6^) (Supplementary Fig. 1). We proposed that genes *phi3T_93* and *phi3T_97* encode the SPβ YopN and YopR functional homologues, respectively. Indeed, deleting *phi3T_97* generated a lytic phage (Extended Data Fig. 1a,b), confirming its role as phi3T’s master repressor. Additionally, and in agreement with a recent report^7^, overexpression of *phi3T_93* or *phi3T_97* in a recipient strain blocked phage reproduction and increased lysogenization (Extended Data Figs. 1c–e and 2c–e), similar to YopN and YopR in SPβ^6^. However, the deletion of *phi3T_93* did not impact the phage titre or number of lysogens after infection (Extended Data Fig. 2a,b).

**Fig. 1:**
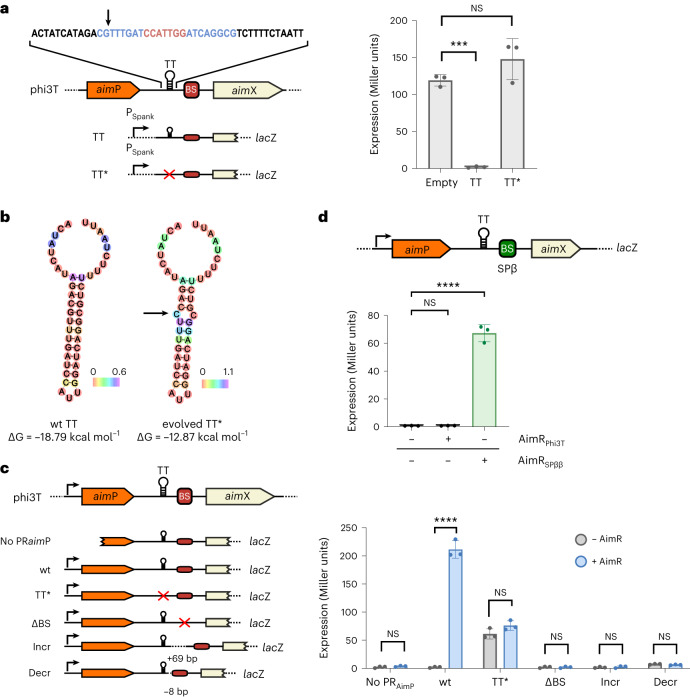
AimR functions as an antiterminator of a transcript that initiates at *aim*P. **a**, Left: schematic view of the regions cloned into the pDG1663+ P_spank_ reporter plasmid and fused to the β-gal reporter gene. The DNA sequence (top) shows the predicted terminator and the regions corresponding to the stems (blue) and loop (red). Right: *β-gal* expression was measured in *B. subtilis* strains carrying the construction after IPTG induction of the P_spank_ promoter. **b**, Predicted minimum free-energy secondary structures of the terminator for the wt and evolved sequence (RNAfold). Colours represent positional entropy in the structure. In **a** and **b**, the black arrow indicates the mutated base. **c**, Left: schematic view of relevant genetic features upstream from *aim*X and of the regions cloned into the reporter plasmid and fused with the *β-gal* gene. **d**, Top: schematic of the genetic features used to characterize cross-activity of AimR antitermination for SPβ and phi3T where the binding site has been swapped. **c**, Right and **d**, Bottom: *β-gal* expression measured in *B. subtilis* strains carrying the integrated reporter plasmid and pDR110 (empty or AimR_phi3T_) or pDR110 (empty, AimR_phi3T_ or AimR_SPβ_) plasmid, respectively, after IPTG induction of the P_spank_ promoter in pDR110. In the schematics: black arrows, promoters; black lollipop symbols, putative transcriptional terminators; TT, transcriptional terminator; TT*, evolved mutated TT; BS, AimR binding site (red, phi3T BS; green, SPβ BS). ∆BS, mutated BS palindromes; Incr, increased distance between the TT and BS; Decr, decreased distance between the TT and BS. Data presented as mean ± s.d. (*n* = 3). In **a** and **d**, an ordinary one-way analysis of variance (ANOVA), followed by Dunnett’s multiple comparisons test, was performed; in **c**, two-tailed *t*-tests were performed. *P* values are indicated above each comparison: ****P < 0.0001; ***P < 0.001; NS, not significant. Source data

**Extended Data Fig. 3:**
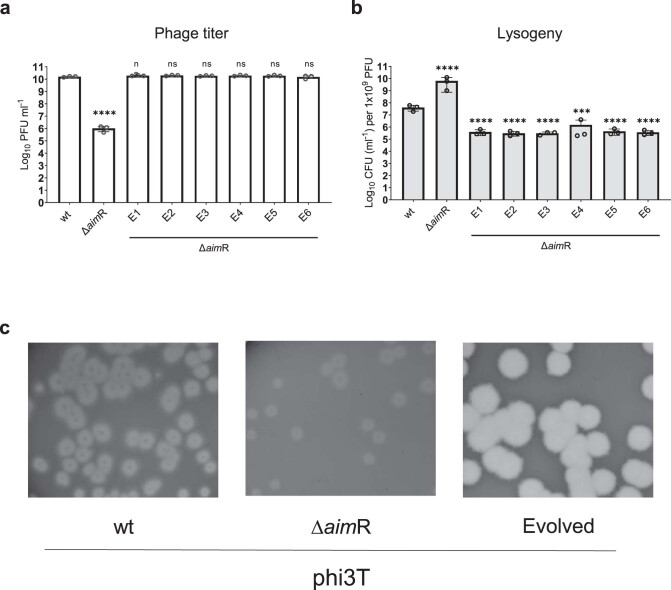
Evolved phi3T Δ*aim*R phages have reduced lysogenisation. Strains lysogenic for phages phi3T wt, Δ*aim*R, and evolved Δ*aim*R phages were MC induced. The number of resulting plaques (**a**) and lysogens **(b)** were quantified using 168 Δ6 as the recipient strain. The results are represented as PFUs ml^−1^ or CFUs ml^−1^ normalised by PFUs ml^-1^ and represented as the CFU of an average phage titer (1×10^9^ PFU ml^-1^). Geometric mean and SD are marked (n = 3). An ordinary one-way ANOVA of log_10_-transformed data followed by Dunnett’s multiple comparisons test was performed to compare mean differences. Comparisons were performed against wt phi3T infecting 168 Δ6. Adjusted p values were as follows: ***p < 0.001; ****p < 0.0001; ns (not significant). **(c)** The resulting plaque morphologies were photographed. Source data

In SPβ, *yop*N and *yop*R were originally identified by evolving the SPβ Δ*aim*R mutant, which is deficient in titre and produces cloudy plaques^6^. We repeated this strategy for the phi3T Δ*aim*R mutant phage. For six evolved phages, from independent experiments, we observed a recovery of phage titre following mitomycin C (MC) induction (Extended Data Fig. 3a). These evolved phages generated lysogens at a significantly reduced level and formed sharp plaques (Extended Data Fig. 3b,c). Surprisingly, none of these evolved phages carried mutations in *phi3T_93* or *phi3T_97*. Instead, they carried the same mutation in a non-coding region between *aim*P and *aim*X (Fig. 1a and Supplementary Table 1), where we identified a putative transcriptional terminator (TT). The compensatory mutation in the evolved phages was predicted (ARNold^8,9^ and RNAfold^10^ software) to impact the stem of the TT and alter its secondary structure (Fig. 1b).

We confirmed that the TT was functional by cloning the region containing the putative TT (or the evolved mutated TT*) with the *aim*X gene transcriptionally fused to the β-galactosidase (β-gal) reporter gene in a plasmid with an isopropyl β-d-1-thiogalactopyranoside (IPTG)-inducible P_Spank_ promoter (Fig. 1a). In the presence of IPTG, the wild-type (wt) TT blocked β-gal (*aim*X) expression but the mutated TT* did not (Fig. 1a). This suggests that the evolved phages restored their function just by disrupting this TT, enabling *aim*X expression from an uncharacterized promoter located upstream of *aim*X.

### AimR is a transcriptional antiterminator

It has been postulated that AimR activates *aim*X expression by binding to the AimR operator located upstream of *aim*X^3^. This region has been characterized, and the structure of AimR bound to DNA has been solved^5,11^. However, we found no putative promoter when analysing (phiSITE tool PromoterHunter^12,13^) the region downstream of the AimR binding site. To test this, a two-plasmid system was used, with one plasmid overexpressing AimR^phi3T^ and a second reporter plasmid carrying the cloned region from the TT (including the AimR binding site) to *aim*X fused to the β-gal reporter. Surprisingly, both in the presence or absence of AimR^phi3T^, no β-gal expression was observed. Introducing the prophage did not result in reporter expression, which excluded the possibility that other phage-encoded factors are required. These results suggested that there is no promoter present for *aim*X in this region and AimR does not induce *aim*X expression as a traditional transcriptional activator. Therefore, we hypothesized that (1) AimR is not a transcriptional activator but an antiterminator and (2) the upstream *aim*P promoter produces the transcript that terminates at the TT in the absence of AimR.

To test this, we cloned *aim*P–*aim*X regions of different lengths that included either the wt or mutated TT (see Fig. 1c for details). In the absence of AimR^phi3T^, no expression of β-gal was observed in plasmids carrying the wt TT or lacking the *aim*P promoter, while expression was observed in the plasmid containing the *aim*P promoter with the mutated TT* (Fig. 1c). In the presence of AimR^phi3T^, expression of the β-gal occurred only in those plasmids that contained the *aim*P promoter (Fig. 1c). Reverse transcription PCR analyses confirmed that *aim*P and *aim*X are part of the same bicistronic operon that initiates at the *aim*P promoter (Supplementary Fig. 2). In addition, mutation of the AimR^phi3T^ binding site in the reporter constructs abolished expression (Fig. 1c), confirming that AimR binding to its specific site is required for antitermination to occur. Altering the distance (increase of 69 bp or decrease of 8 bp) between the TT and the AimR binding site also abolished AimR function, supporting the function of AimR as an antiterminator instead of a transcriptional activator (Fig. 1c).

**Extended Data Fig. 4:**
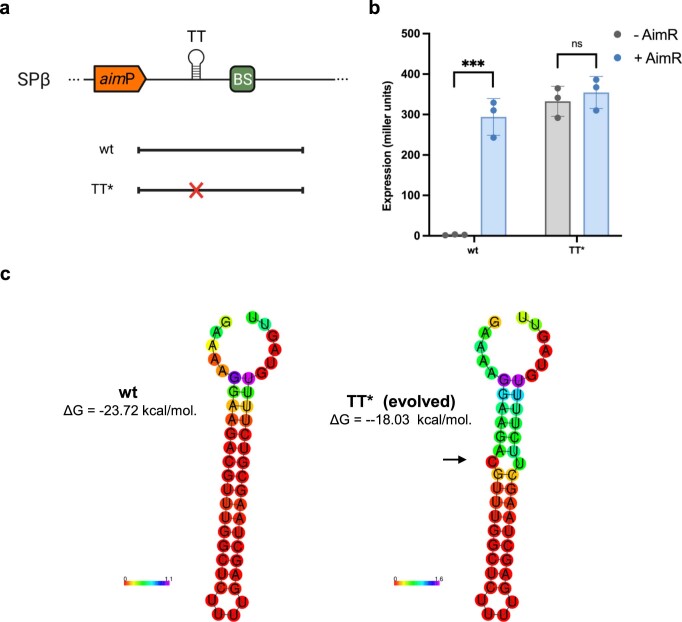
AimR is an antiterminator in phage SPβ. **(a)** Schematic view of the regions of SPβ DNA cloned into the pDG1663+ P_Spank_ reporter plasmid. TT*: mutated transcriptional terminator. **(b)** *B. subtilis* strains carrying pDR110 (empty or AimR^SPβ^) and the reporter plasmids were grown to OD_600_ 0.2. IPTG was added for induction of the P_Spank_ promoter in the pDR110 plasmid (empty or AimR). β-gal expression was measured using the Miller method. Data presents the mean and SD (n = 3). Two-tailed t-tests were performed to compare mean differences; p values indicated were as follows: ***p = 0.0004; ns (not significant). replicates. **(c)** Predicted minimum free energy secondary structures of the terminator for the wt and TT* (evolved) sequence according to RNAfold. Colours are representative of positional entropy in the structure. Black arrow indicates the mutated bp. Source data

In a similar way, we confirmed that AimR from SPβ (AimR^SPβ^) also functions as an antiterminator, preventing transcript termination of a TT identified upstream of the AimR^SPβ^ binding site (Extended Data Fig. 4a,b). We also obtained 8 additional SPβ Δ*aim*R evolved phages with restored capacity to form normal plaques. Three of them contained a single bp mutation in the TT upstream of the AimR^SPβ^ binding site (Supplementary Data Table 1), which inactivated its functionality (Extended Data Fig. 4c). Finally, by swapping the AimR binding sites of phi3T and SPβ in a reporter construct carrying the phi3T region from *aimP* promoter to *aimX* (see scheme in Fig. 1d), we confirmed that the mechanism of antitermination is conserved in both phages and requires the interaction of AimR with their cognate DNA-binding sites^5,11^ (Fig. 1d).

In summary, our results challenge the current understanding of how the arbitrium system works, suggesting that *aim*P and *aim*X form a bicistronic operon regulated by an AimR antitermination mechanism.

### phi3T *aim*X encodes a small peptide

**Fig. 2:**
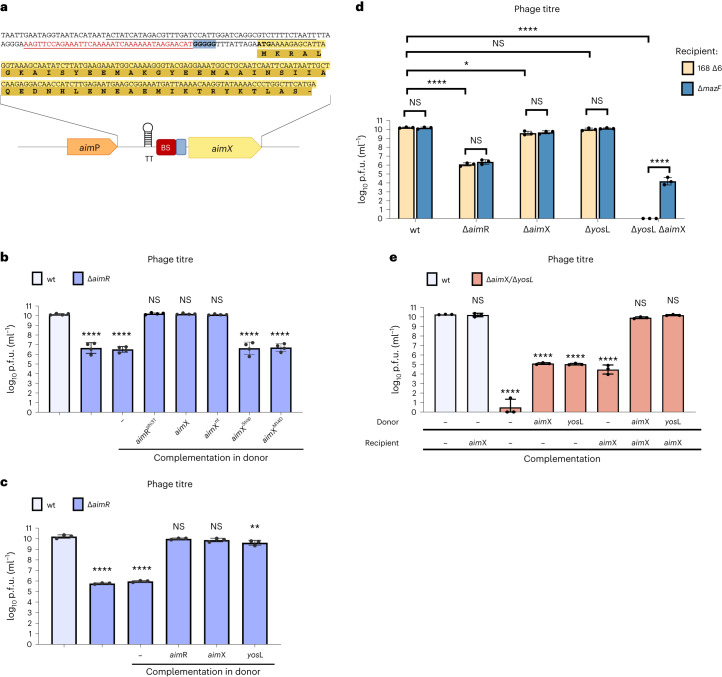
AimX is a peptide showing functional similarity to YosL. **a**, Representation of the DNA region located between *aim*P and *aim*X. The TT is underlined in black. The AimR binding site (BS) is underlined in red. The Shine–Dalgarno region is highlighted in blue. The AimX coding sequence and the encoded protein sequence are highlighted in light and dark yellow, respectively, with the start codon in bold. **b**,**c**, Strains lysogenic for phages phi3T wt, Δ*aim*R and Δ*aim*R complemented (*amy*E::P_spank_−) with aimR; different versions of the *aim*X (**b**) or *yos*L (**c**) gene were MC induced. Resulting phages were quantified using 168 Δ6 as the recipient strain. **d**, 168 Δ6 strains lysogenic for phi3T wt and mutant (Δ*aim*R, Δ*aim*X, Δ*yos*L, double Δ*aim*X/Δ*yos*L) were MC induced. Resulting phages were quantified using 168 Δ6 or 168 Δ6 Δ*maz*F mutant as recipient strains. **e**, Donor 168 Δ6 strains lysogenic for phages phi3T wt or Δ*aim*X/Δ*yos*L and carrying empty vector (–) or expressing *aim*X or *yos*L were MC induced. Resulting phages were quantified using 168 Δ6 *amy*E::P_Spank_-empty (–) or 168 Δ6 *amy*E::P_Spank_-*aim*X as recipient strains. Results are presented as p.f.u.s ml^−1^ and geometric mean and geometric s.d. are shown: (**b**) *n* = 4, (**c**) *n* = 3 (**d**) *n* = 3 and (**e**) *n* = 3. Values below the detection limit of 10 p.f.u.s ml^−1^ were assigned a value of 1 and marked on the axis. An ordinary one-way ANOVA of log_10_-transformed data, followed by Dunnett’s multiple comparisons test, was performed to compare mean differences between titres in **b**, **c** and **e**. Comparisons were performed against wt phi3T. A two-way ANOVA of log_10_-transformed data, followed by Tukey’s multiple comparisons test, was performed to compare mean differences in titres in **d**. Adjusted *P* values: ****P < 0.0001; **P < 0.005; *P < 0.05. Source data

While it was assumed that *aim*X encodes a small antisense RNA (sRNA)^3^^,4^, the fact that *aim*X is part of a bicistronic transcript suggests that it may encode a peptide, a possibility already considered in the original article reporting the arbitrium system^3^. In the phi3T genome, a 51-amino-acid-long open reading frame, phi3T_91, was annotated in the *aim*X locus, and our in silico analysis identified all the elements required for translation (Fig. 2a).

To test this, we established an approach to verify *aim*X functionality in vivo. Since the role of AimR seems to be to allow *aim*X expression by preventing termination, we expressed different versions of *aim*X from an ectopic chromosomal locus and checked whether they restored the severely affected induction of the Δ*aim*R mutant prophage^6,14^. The wt *aim*X and a version with an altered DNA sequence (*aim*X^nt^), but encoding the same protein, successfully complemented the Δ*aim*R mutant phage, both in the donor (restored efficient prophage induction) and recipient cells (restored plaque morphology). In contrast, a construct with a stop codon in the fifth amino acid (*aim*X^Stop^) did not complement the Δ*aim*R mutation (Fig. 2b and Supplementary Fig. 3a–c). We also expressed AimX heterologously in *E. coli* (fused to an N-terminal histidine tag) and purified the product, yielding a soluble monomeric protein in solution (Supplementary Fig. 4a,c). This supports the idea that *aim*X encodes a peptide and not a regulatory RNA in phi3T.

### AimX interacts with phage phi3T_93 and host MazF

**Fig. 3:**
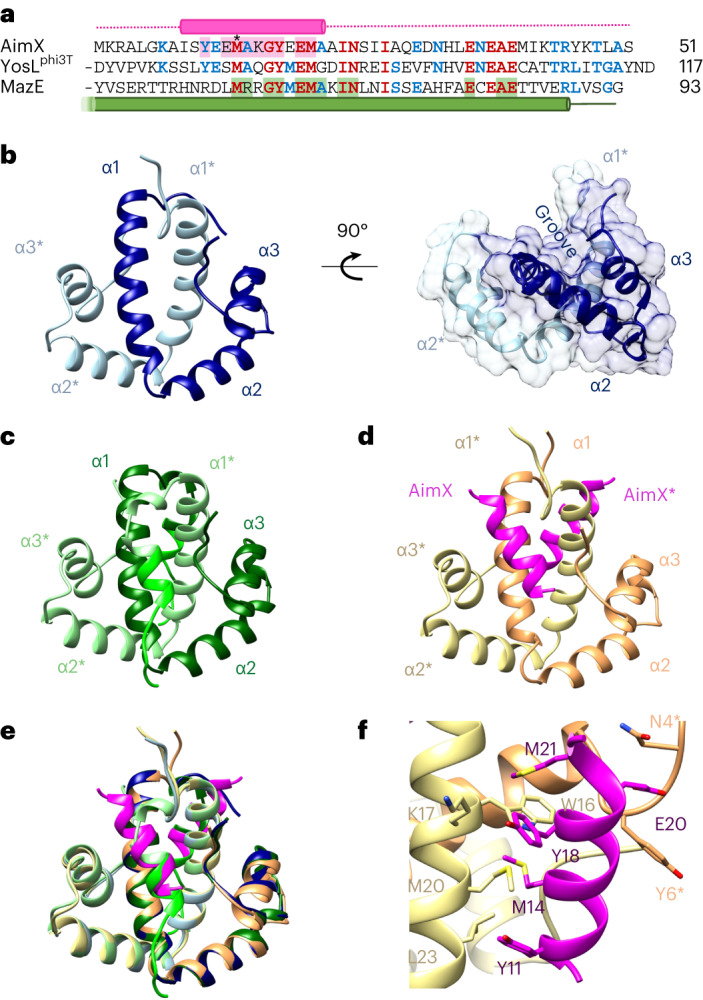
Molecular and structural characterization of arbitrium–MazE/F net of interactions. **a**, Sequence alignment for the homologous region of AimX, YosL^phi3T^ and MazE. Residues conserved in two or three of these sequences are highlighted in blue or red, respectively. Structural elements are shown for AimX and MazE in magenta and green, respectively. The AimX and MazE residues involved in contacts with their partners phi3T_93 and MazF are highlighted in pink and green background, respectively. The mutated methionine in AimX that abolishes phi3T_93 binding is pointed with a star. **b**, Ribbon representation of phi3T_93 (left) and side view of phi3T_93 surface (right). Each phi3T_93 monomer is depicted in a different blue shade and helices are numbered (* denotes second monomer). **c**, Ribbon representation of His-phi3T_93 structure. Each monomer is shown in a different shade of green. Residues belonging to the His-tag linker depicted in fluorescent green in both monomers. **d**, Ribbon representation of AimX-phi3T_93 complex with phi3T_93 monomers coloured in different shades of yellow and AimXs in magenta. **e**, Superimposition of AimX-phi3T_93 complex and His-phi3T_93 structures, following the same colour pattern as in **d** and **c**, respectively. **f**, Close-up view of the residues involved in phi3T_93 and AimX interaction. Interacting residues are labelled. Source data

To explore AimX function and identify potential targets, we performed a sequence similarity search. Apart from multiple AimX homologues, BLASTp^15^ analyses showed two additional candidates sharing >50% identity with a region of AimX: (1) MazE, a host-encoded antitoxin that binds to its cognate MazF toxin inactivating its ribonuclease activity^16^ and (2) YosL, encoded by both SPβ and phi3T. MazE and YosL have similar sizes (100–130 residues) and show sequence similarity with AimX in their C-terminal regions (Fig. 3a). Importantly, this region of MazE is involved in binding to the MazF toxin^16^. Therefore, while AimX might bind to and modulate the function of one of the known lysogeny-promoting phi3T proteins (phi3T_93 or phi3T_97), the similarity of AimX to MazE suggested another possible target, the toxin MazF.

To test the potential binding partners of AimX, we used biolayer interferometry (BLI) assays, which showed that AimX does not bind to the master repressor phi3T_97 but binds to both MazF and phi3T_93. Interestingly, the affinity is more than twice as high for MazF than for phi3T_93 (Extended Data Table 1). In addition, MazF has five times more affinity for AimX than for MazE, its cellular partner (Extended Data Table 1). In vitro competition assays confirmed this preference, showing that AimX rapidly displaces MazE from its complex with MazF (Supplementary Fig. 5). The affinity differences of both complexes are mainly due to a decrease in dissociation constant (*k*_off_), suggesting a more stable AimX–MazF complex with a potential regulatory function in the lysis–lysogeny decision (Extended Data Table 1).

Next, we analysed whether MazE and YosL^phi3T^ are also able to interact with phi3T_93 and MazF. In agreement with a recent study^7^, MazE also binds to phi3T_93, which potentially results in an increased amount of active MazF^7,16^. MazE has almost 15 times higher affinity for phi3T_93 than for its cellular target MazF (Extended Data Table 1). Surprisingly, while YosL^phi3T^ does not bind to the phage phi3T_93, it binds to the host MazF. MazF binds to YosL^phi3T^ with a higher affinity than with AimX or MazE, a similar affinity to that of the MazE–phi3T_93 complex (Extended Data Table 1). Similar results were obtained with YosL from phage SPβ (YosL^SPβ^), confirming the YosL binding specificity of MazF (Extended Data Table 1). These results suggest that a common region of MazE, AimX and YosL is responsible for recognizing MazF, thus promoting AimX and YosL to putative antitoxins.

### In vivo characterization of YosL

Our results suggested that the main role of AimR is regulating AimX expression, and overexpression of AimX from an ectopic chromosomal locus restored efficient Δ*aim*R prophage induction (Fig. 2b,c). However, induction of a Δ*aim*X mutant prophage resulted in a titre similar to that of the wt phage, unlike the severely reduced titre of the Δ*aim*R prophage^14^ (Fig. 2b). One possible explanation is that AimR may control (directly or indirectly) the expression of another protein, whose function at least partially overlaps with the function of AimX. On the basis of our previous results, we hypothesized that YosL was a candidate.

Indeed, complementing the Δ*aim*R phi3T mutant by overexpressing *yos*L upon prophage induction restored high titre levels (Fig. 2c). We also attempted to complement the recipient strain during infection; however, we observed that overexpression of *yos*L prevented normal cell growth. During induction experiments, complementation was possible since we added IPTG and MC simultaneously, once the cells have already reached the desired density. Next, we generated a phi3T Δ*yos*L and a Δ*aim*X/Δ*yos*L double mutant. No defect in phage titre was observed after the Δ*yos*L prophage induction (Fig. 2d); however, the Δ*aim*X/Δ*yos*L prophage was severely affected in its ability to generate infective particles, even more than the Δ*aim*R mutant (Fig. 2d,e), and the cultures lysed badly upon induction. Simultaneous complementation of the Δ*aim*X/Δ*yos*L mutant in the donor strain (with either *aim*X or *yos*L) and in the recipient (*aim*X) led to full titre recovery, while complementation in only recipient or donor resulted in partial titre recovery (Fig. 2e). Therefore, these proteins impact both the induction and infection processes and have, to some extent, an overlapping function.

### MazF impacts the lysis–lysogeny outcome in phi3T

**Fig. 4:**
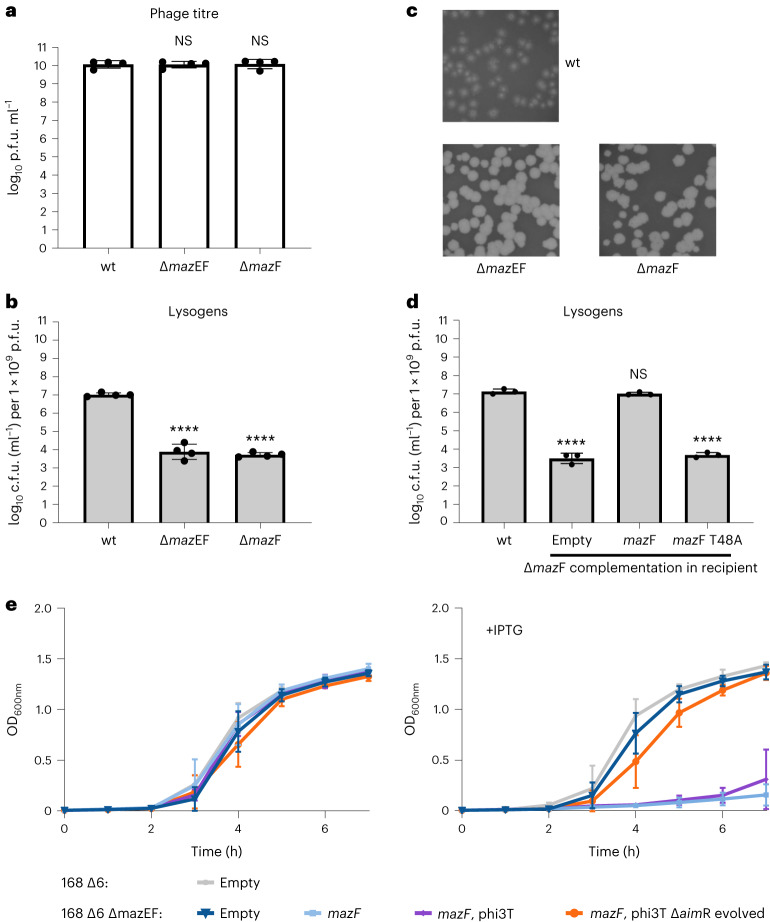
MazF activity is important for efficient lysogeny of phi3T phage. 168 Δ6 strain lysogenic for phi3T was MC induced. **a**,**b**, The number of resulting phages (**a**) and lysogens (**b**) were quantified using 168 Δ6 (wt) or mutated 168 Δ6 (ΔmazEF, ΔmazF) recipient strains. Data represent the geometric mean and geometric s.d. (*n* = 4). **c**, The resulting plaque morphologies were photographed. **d**, To test for complementation of the 168 Δ6 Δ*maz*F strain, phi3T was used to infect 168 Δ6 (wt) or 168 Δ6 Δ*maz*F-expressing amyE::P_Spank_-empty/*maz*F/*maz*F^T48A^ (mutant of mazF with no RNAse activity) as the recipient strains. Data represent geometric mean and geometric s.d. (*n* = 3). In **a**, results are shown as p.f.u.s ml^−1^, and in **b** and **d** as c.f.u.s ml^−1^ normalized by p.f.u.s ml^−1^ and represented as the c.f.u. of an average phage titre (1 × 10^9^ p.f.u.s ml^−1^). **e**, Growth curves (OD_600_ readings) for strains carrying different IPTG-inducible expression vectors (amyE::P_Spank_) and lysogenic phages that were grown without inducer (left) or with IPTG (right). Mean ± s.d. (*n* = 3) are presented. Strains used: wt 168 Δ6 with empty vector (grey); 168 Δ6 Δ*maz*EF with empty vector (dark blue), expressing *mazF* (light blue), expressing *mazF* and lysogenic for wt phi3T prophage (purple), expressing *mazF* and lysogenic for evolved Δ*aim*R phi3T prophage (orange). An ordinary one-way ANOVA of log_10_-transformed data, followed by Dunnett’s multiple comparisons test, was performed to compare mean differences in titre numbers in **a**, **b** and **d**. Comparisons were performed against wt phi3T infecting 168 Δ6. Adjusted *P* values: ****P < 0.0001. Source data

MazF is an RNA endoribonuclease^17^ during normal growth, forming a stable complex with its cognate antitoxin, MazE^16,17^. However, if MazE is degraded or sequestered, MazF is released and cleaves cellular mRNAs to inhibit protein synthesis, leading to growth arrest^16^. The fact that both AimX and YosL bind to MazF suggested that this toxin may perform a central role in the life cycle of phi3T. The titre obtained when infecting the Δ*maz*F or Δ*maz*EF mutant strains with phi3T was identical to that obtained with the wt recipient strain (Fig. 4a); however, the number of lysogens was severely reduced in the absence of MazF (Fig. 4b) and the plaques produced were significantly sharper and larger (Fig. 4c). These observed differences were reverted by expression of the wt MazF in the recipient cells, but not after expression of the MazF T48A mutant which has no RNAse activity^16^ (Fig. 4d). Taken together, these results indicated that MazF’s activity impacts the lysis–lysogeny result by promoting lysogeny and/or inhibiting the lytic cycle.

A recent study suggested that the active MazF toxin suppresses phi3T phage’s lytic propagation, possibly by an abortive infection mechanism^7^. Since AimX and YosL favour the lytic cycle of the phage, the formation of AimX–MazF or YosL–MazF complexes may block MazF activity. To test this, we introduced an IPTG-inducible *maz*F gene into the Δ*maz*EF strain, deprived of its natural antitoxin protein, MazE. Next, we lysogenized this strain with the wt phi3T phage or evolved phage carrying the double *aim*R/TT mutation, which constitutively expresses AimX. As predicted, toxicity of the IPTG-induced MazF was blocked in the presence of the evolved prophage expressing AimX, enabling bacterial growth (Fig. 4e).

**Extended Data Fig. 5:**
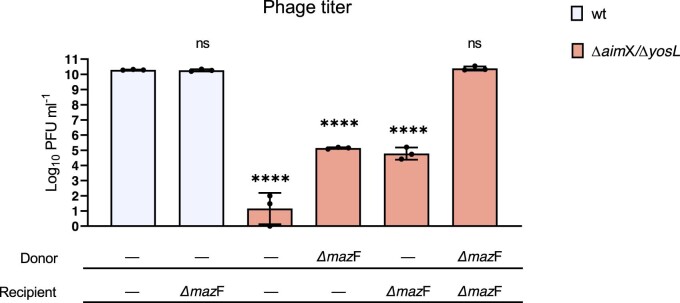
When MazF is present in donor or recipient strains, there is a reduced number of plaques resulting from a phi3T Δ*aim*X Δ*yos*L mutant infection. Strains lysogenic for phi3T wt or phi3T mutant Δ*aim*X/Δ*yos*L, were MC induced from different donor strains: 168 Δ6 (indicated as –) and 168 Δ6 Δ*maz*F. The number of resulting plaques was quantified using 168 Δ6 or 168 Δ6 Δ*maz*F mutants as recipient strains. Geometric mean and SD (n = 3) of the number of resulting plaques (PFUs ml^−1^) are presented. An ordinary one-way ANOVA of log_10_ -transformed data followed by a Dunnett’s multiple comparisons test was performed to compare mean differences. Comparisons were performed against wt phi3T infecting 168 Δ6. Adjusted p values indicated are as follows: ****p < 0.0001; ns (not significant). Values below the detection limit of 10 PFUs ml^−1^ were assigned value 1 and marked on the axis. Source data

We hypothesized that the Δ*yos*L/Δ*aim*X double mutant phage produced a very low titre due to its inability to block MazF activity, which leads to suppression of lytic propagation. Indeed, the mutant phage formed more and sharper plaques in Δ*maz*F recipient strain. In addition, a Δ*maz*F strain lysogenized with the Δ*aim*X/Δ*yos*L prophage lysed well after induction and produced an increased titre compared with the same prophage induced from the strain containing MazF. To fully restore the wt titre level, MazF had to be absent in both donor or recipient, indicating that both induction and infection are impacted by these interactions (Extended Data Fig. 5). Together, these results indicate that AimX or YosL needs to block MazF activity for the lytic cycle to proceed.

### Structural characterization of the AimX–phi3T_93 complex

On the basis of these results, we propose that AimX, YosL, phi3T_93, MazE and MazF are involved in a network of mutually exclusive interactions essential for the lysis–lysogeny decision. Therefore, we attempted the structural characterization of its components. The structures of MazF in complex with MazE (PDB 4ME7) or with a target RNA (PDB 4MDX) have already been reported^16^. We obtained the structure of phi3T_93 alone (wt and its N-terminal His-tagged version; His-phi3T_93) and in complex with AimX (Extended Data Table 2). In all structures, phi3T_93 shows an identical all-helices fold composed of a longer N-terminal helix (α1), followed by two shorter helices (α2 and α3) connected by a long unstructured loop (Fig. 3b–d) forming a V-like shape. The α1 helices of two phi3T_93 monomers interact, resulting in a W-shaped tight and globular dimer that displays two shallow grooves, one across each side of the dimer molecule (Fig. 3b). Size exclusion chromatography coupled to multi-angle light scattering (SEC–MALS) analysis confirmed the phi3T_93 dimeric organization in solution (Supplementary Fig 4b,c).

**Extended Data Fig. 6:**
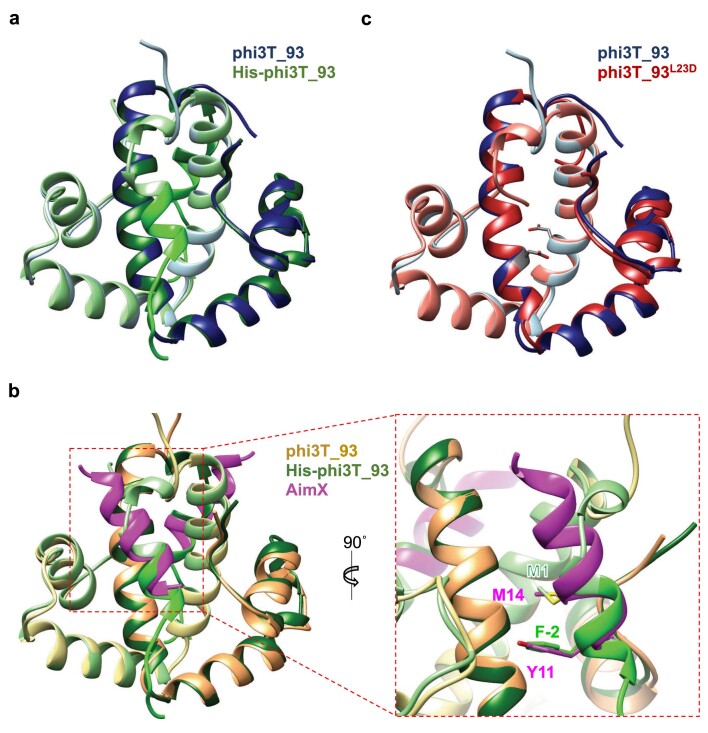
phi3T_93 structures superimposition. Superimposition of the ribbon represented crystal structures of phi3T_93 (shades of blue) and His-phi3T_93 (shades of green) **(a)**, phi3T_93, His-phi3T_93 and AimX-phi3T_93 complex **(b)** and phi3T_93 and phi3T_93^L23D^**(c)**. Note that phi3T_93 and His-phi3T_93 dimers are coloured in shades of blue and green respectively. Residues belonging to the linker with the His-tag are coloured in fluorescent green for both monomers. AimX is coloured in magenta whereas phi3T_93 bound to AimX is coloured in shades of yellow. phi3T_93^L23D^ dimer is coloured in shades of red, whereas the mutated residue is displayed in dark grey.

**Extended Data Fig. 7:**
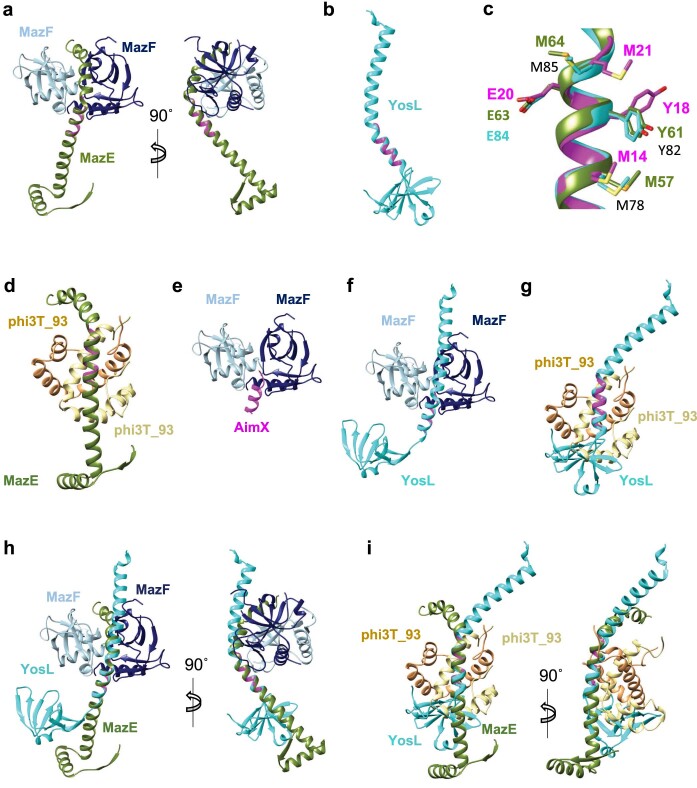
Experimental and *in silico* models for the net of mutually exclusive interactions. **(a)** Experimental complex (PDB 4ME7) of MazF (blue shades) and MazE (green) superimposed to the homologous region of AimX (magenta). (**b**) Alphafold model for YosL^phi3T^ (cyan) superimposed to the homologous region of AimX (magenta). (**c**) Close view of the homologous region of AimX, MazE and YosL^phi3T^ showing the conserved residues involved in partner recognition in sticks and labelled. Models of the complexes (**d**) MazE-phi3T_93, (**e)** MazF-AimX, (**f)** YosL-MazF and (**g)** phi3T_93-YosL generated by the superimposition of the homologous alpha helix. **(h)** Two orthogonal views of the superimposition of YosL to the MazF-MazE complex show no steric problems for YosL-MazF complex formation. (**i**) Two orthogonal views for the superimposition of potential phi3T_93-YosL and phi3T_93-MazE complexes revealing steric hindrances for the N-terminal domain of YosL with phi3T_93 dimer but not for MazE. Note that in all the models AimX is depicted in magenta to show the relative position in the complex against the rest of the components.

The structure of the AimX–phi3T_93 complex showed that a phi3T_93 dimer binds two independent AimX molecules that are accommodated in the shallow grooves (Fig. 3d,e). Only a short fragment of AimX (residues 8–22; 30% of the protein), which folds as a 3-turn helix, is visible in the AimX–phi3T_93 crystal (Fig. 3a,d). The main contacts between AimX and phi3T_93 are nucleated by the projection of AimX M14 and M21 towards the hydrophobic bottom of the phi3T_93 groove generated by the side chains of K13, K17, W16, M20 and L23, pointing to these hydrophobic interactions as key for phi3T_93–AimX recognition (Fig. 3f and Supplementary Table 2). Additional polar and hydrophobic interactions stabilize the complex (Supplementary Table 2). Interestingly, in the His-phi3T_93 structure, the N-terminal His-tag folds as helices and are also accommodated in the dimer grooves replicating the AimX contacts (Fig. 3c,e, Extended Data Fig. 6b and Supplementary Table 2), supporting the idea that the phi3T_93 grooves are specifically designed to accommodate a short helix. The portion of AimX visible in the structure corresponds to an area of high sequence similarity with MazE and YosL (Fig. 3a), explaining why all these three proteins bind to MazF. In MazE, this region also folds as a helix and Alphafold also proposed a similar fold for YosL^phi3T^ (Extended Data Fig. 7a,b). Despite the structural differences between phi3T_93 (all alpha fold) and MazF (mainly beta fold), both AimX and MazE use an identical recognition mechanism by introducing a helix in the groove in the dimer’s interface (Fig. 3b and Extended Data Fig. 7a). Structural alignment showed identical residues in the helix of AimX (M14, Y18, E20 and M21 for AimX) and MazE involved in the interactions with the corresponding partners, and YosL^phi3T^ also presents identical residues in these positions (Fig. 3a and Extended Data Fig. 7a–c). Models of the putative AimX–MazF, YosL–MazF, MazE–phi3T_93 and YosL–phi3T_93 complexes showed that these key conserved residues occupy identical spatial arrangements allowing them to mimic interactions with their alternative partners (Extended Data Fig. 7d–g). In agreement with the BLI results (Extended Data Table 1), no steric hindrance was observed in the AimX–MazF, YosL–MazF and MazE–phi3T_93 models; however, for the YosL–phi3T_93 complex, steric clashes were observed between the YosL N-terminal domain and the phi3T_93 dimer (Extended Data Fig. 7d–i).

### In vitro and in vivo validation of the interaction network

Our in silico complexes indicate that the AimX M14 residue plays an essential role in phi3T_93 and MazF recognition and substituting for a charged residue in that position should prevent AimX function (Fig. 3a,f and Extended Data Fig. 7c). Indeed, while the AimX^M14D^ mutant showed similar yield and behaviour in solution as the wt protein (Supplementary Fig 4a,c), BLI analysis showed no binding of AimX^M14D^ to either phi3T_93 or MazF (Extended Data Table 1). As expected, the expression of the AimX^M14D^ mutant, unlike that of the wt AimX, did not complement the defective induction of the phi3T Δ*aim*R prophage (Fig. 2b).

**Fig. 5:**
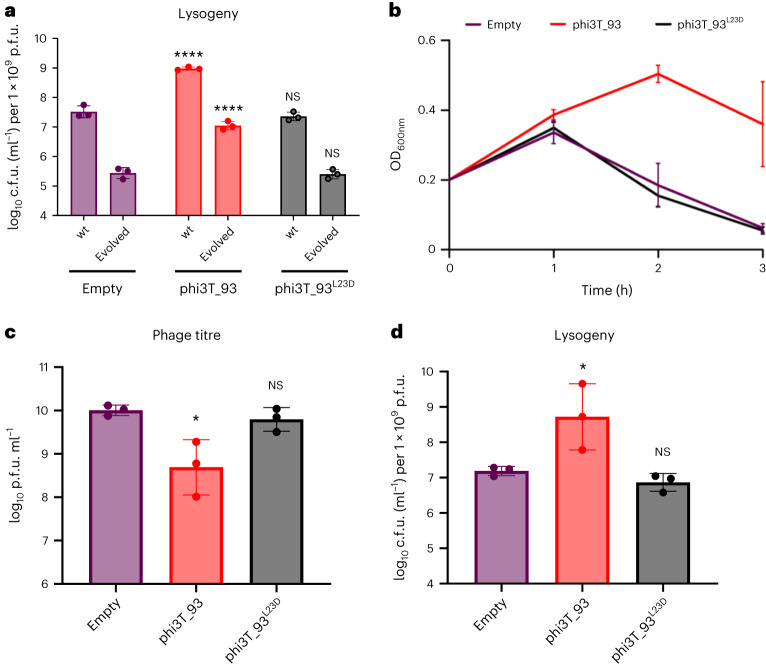
phi3T_93 promotes lysogeny. **a**, Strains lysogenic for phi3T wt and evolved Δ*aim*R phages were MC induced. The number of lysogens were quantified using 168 Δ6 *amy*E::P_Spank_-empty or 168 Δ6 *amy*E::P_Spank_-phi3T_93 (wt/L23D) as the recipient strains. **b**–**d**, *B. subtilis* strains either devoid of (purple) or expressing wt phi3T_93 (red) or phi3T_93^L23D^ (grey) were infected with phage phi3T wt at an MOI of 1:10 (phage:bacteria). For all infection experiments, growth (OD_600_) readings (**b**), phage titres (**c**) and the relative number of lysogens (**d**) were determined after 3 h. Results are presented as p.f.u.s ml^−1^ in **c**, or c.f.u.s ml^−1^ normalized by p.f.u.s ml^−1^ and presented as the c.f.u. of an average phage titre (1 × 10^9^ p.f.u. ml^−1^) in **a** and **d**. In **a**, **c** and **d**, geometric mean and geometric s.d. are shown (*n* = 3), and in **b**, mean ± s.d. are shown (*n* = 3). An ordinary one-way ANOVA of log_10_-transformed data, followed by Dunnett’s multiple comparisons test, was performed to compare mean differences in titre or relative lysogen numbers. Comparisons were performed against results for the same phage (wt phi3T or evolved) infecting 168 Δ6 or 168 Δ6 *amy*E::P_Spank_-empty strain. Adjusted *P* values: ****P < 0.0001; *P < 0.05. Source data

Next, we produced phi3T_93^L23D^ by replacing a hydrophobic residue that interacts with the AimX M14 for a negatively charged one. The new charged residue had no impact on the protein integrity and quaternary organization as confirmed by solving its crystal structure and SEC analyses (Extended Data Fig. 6c and Table 2, and Supplementary Fig. 4b,c). BLI analysis showed no binding of phi3T_93^L23D^ to AimX and a strong reduction (two orders of magnitude) in its affinity for MazE (Extended Data Table 1). We hypothesize that phi3T_93 promotes lysogeny by binding AimX and MazE, reducing the amount of antitoxins that can block MazF activity. Therefore, we used the evolved phi3T Δ*aim*R TT-mutant which expresses AimX constitutively and as a result produces less lysogens (Extended Data Fig. 3b). Overexpression in the recipient cell of phi3T_93, but not of the phi3T_93^L23D^, restored the ability of the evolved phage to produce lysogens (Fig. 5a). This confirmed that phi3T_93–AimX interactions are important for the lysis–lysogeny decision. In addition, strains expressing phi3T_93, unlike those expressing phi3T_93^L23D^, blocked the lytic cycle and promoted lysogeny of the wt phi3T (measured by plaque and colony counts 3 h after infection; Fig. 5b–d). These results validate both the structural complexes and the common mechanism of recognition observed for AimX–phi3T_93 and MazE–MazF, which are driven mainly by the insertion of a hydrophobic helix into the grooves of the dimers of phi3T_93 and MazF. Given the conservation of residues in the proposed interacting helix of YosL, this model could also be extended to complexes of this putative antitoxin with MazF.

Collectively, our in vitro and in vivo results confirm the intricate network of mutually exclusive, and to some extent functionally redundant, interactions involving the phage arbitrium system and host MazEF toxin–antitoxin systems in the lysis–lysogeny decision.

## Discussion

**Extended Data Fig. 8:**
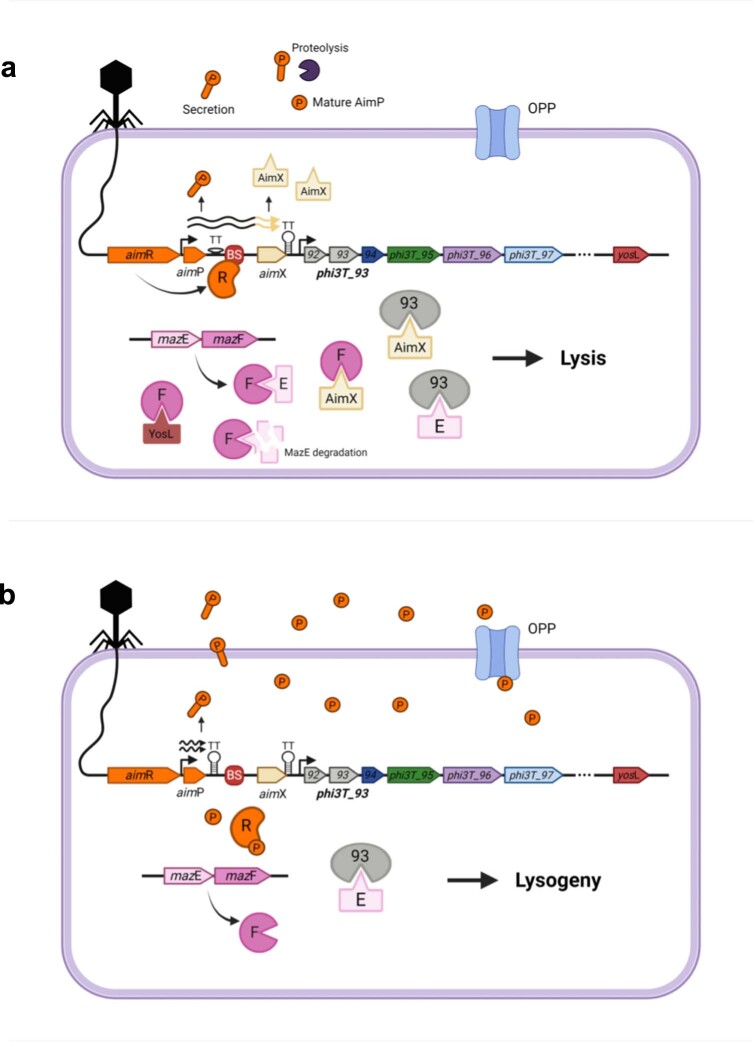
Model for the arbitrium system. **(a)** In early stages of the infection process or after induction of the SOS response, when the concentration of AimP is low, AimR binds to its cognate DNA-binding site (BS), acting as an antiterminator that allows the expression of *aim*X as part of a bicistronic operon starting from the *aim*P promoter. The AimX peptide binds to the toxin MazF and to phi3T_93, preventing its binding to the antitoxin MazE, freeing MazE to bind to MazF. Additionally, YosL binds to MazF. All these interactions result in blocking the function of MazF and promote the phage lytic cycle. **(b)** During late stages of the infection, AimP accumulates inside the bacteria and binds to AimR, inhibiting its function as an antiterminator. When this occurs, the mRNA transcript that starts at *aim*P will stop at the transcriptional terminator (TT) localised upstream *aim*X, preventing its expression. In absence of AimX, phi3T_93 will bind to MazE, liberating MazF whose activity promotes lysogeny by an unknown mechanism. Model created with BioRender.com.

Arbitrium communication systems are highly diverse and are present in numerous phages and other mobile genetic elements^4^. Despite this diversity, it has been widely assumed that they share a conserved mode of action: AimR is a transcriptional regulator controlling the expression of *aim*X, which then directly inhibits the phage master repressor using an uncharacterized *cis*-antisense mechanism^3^. Our study challenges the roles associated with the arbitrium players and introduces additional ones, both for the phage (phi3T_93, YosL) and for the host (MazEF). We have demonstrated that MazF is key in determining whether phi3T will enter the lytic or the lysogenic cycle, and that the major function of the arbitrium system in phi3T is to control MazF activity. Our model assumes that blocking MazF activity is essential for the lytic cycle to proceed. MazF has been described as a defence mechanism that blocks phage infection, so we expect that upon infection and in response to cellular damage, MazE is degraded and MazF released^18,19^. Therefore, at early stages of infection, AimR inhibits the function of a TT located between *aim*P and *aim*X, allowing simultaneous expression of the *aim*P–*aim*X transcript (Extended Data Fig. 8). AimX, as well as another phage protein, YosL, bind to MazF and inactivate its function. AimX can also bind to phi3T_93, preventing it from binding to MazE which would release MazF. Once AimP concentration increases in later infection stages, it will bind to AimR, inactivating its function. AimX will not be produced, allowing phi3T_93 to bind MazE, thus liberating MazF (Extended Data Fig. 8). MazF then promotes lysogeny by an uncharacterized mechanism, possibly involving preferential cleaving of transcripts involved in the lytic cycle^20^. Intriguingly, deletion of phi3T_93 did not visibly impact phage titre or lysogen numbers. This, in agreement with a recent paper^20^, indicates that there might be functional redundancy not only between AimX and YosL, but also between another protein and phi3T_93, suggesting an even more complex regulatory network. This network of mutually exclusive interactions seems to be based on two main concepts: redundancy to ensure a fine-tuning control of the process and molecular mimicry which uses a conserved mechanism of recognition of the MazEF toxin–antitoxin system that the phage mimics with AimX, YosL and phi3T_93. The precise regulation of the lysis–lysogeny decision appears to rely on a complex balance among all of these components, encompassing differences in their binding strength, expression levels and potentially the timing of their expression.

Interestingly, phi3T not only suppresses the host’s defence systems, but possibly exploits it for its own benefit. Since toxin–antitoxin systems can be indicators of cellular stress^21^, involving them in the lysis–lysogeny decision may help the phage sense the physiological state of the host, beyond the classical SOS response required for induction. The connection between arbitrium and SOS pathways remains unknown in phi3T and may involve other genes encoded in the *phi3T_92*–*phi3T_97* operon^3,4,22^.

Another question is why *aim*X expression depends on an antiterminator and not a transcriptional activator? Potentially, this could accelerate the lysis–lysogeny switch by linking, via AimR, signalling (AimP) and effector (AimX) molecules with antagonistic functions. Interestingly, many SPbeta phages seem to encode an sRNA, not an AimX protein^3,4,22^. Possibly, this sRNA may act as a preferential substrate of MazF, blocking the activity of this toxin on other targets by competition and thus enabling the lytic cycle. In addition, we have previously demonstrated that AimR binds to different binding sites across the bacterial genome^5^. Therefore, a potential AimR antiterminator regulon might control the lysis–lysogeny decision in arbitrium phages.

The arbitrium system was the first report of a viral communication system regulating lysis–lysogeny decisions. However, it is now clear that communication between phages and/or with their host for decision making is a more generalized feature as observed in *Vibrio* phages with quorum-sensing receptors that regulate lysis–lysogeny in response to bacterial host inducers, or in examples of bacterial quorum-sensing molecules inducing the lytic cycle in different phages^23,24^. Our findings reveal a set of additional regulatory layers related to the lysis–lysogeny decision pathway, which must integrate different signals from the phage, host and the mutual environment to optimize the phage’s survival. Since arbitrium is present not only in phages but also in other mobile genetic elements that infect both pathogenic and non-pathogenic bacteria, unravelling these complex networks of interactions is crucial to understanding how these phages, mobile genetic elements and bacteria evolve.

Finally, we would like to mention that in a parallel study we completed the characterization of the six-gene operon that contains the *yopMNOPQR* genes in phage SPβ and the *phi3T_92* to *phi3T_97* genes in phage phi3T^22^. Based on its function, we have renamed this operon as the ‘SPbeta phages repressor operon’ (*sro*) and proposed that the genes in the operon now be referred to as *sroABCDEF*^SPβ^ or *sroABCDEF*^phi3T^.

## Methods

### Bacterial strains and growth conditions

All bacterial strains used in this study are listed in Supplementary Table 3. *B. subtilis* and *E. coli* strains were routinely grown at 37 °C in liquid broth (LB Miller or LB Lennox, respectively) with shaking at 200 r.p.m. and 180 r.p.m., or plated into corresponding LB media with 1.5% (w/v) bacteriological agar plates. When required, antibiotics were utilized at the following concentrations: erythromycin (1 μg ml^−1^ or 5 μg ml^−1^), kanamycin (10 μg ml^−1^), ampicillin (100 μg ml^−1^), tetracycline (10 μg ml^−1^) or spectinomycin (100 μg ml^−1^).

### Strain construction

Phages SPβ (NC_001884) and phi3T (KY030782) were used in this study. To generate the conditional *phi3T_97* deletion mutant in phage phi3T, we first introduced into the Δ6 phi3T *phi3T_5*::*kan* strain a copy of the *phi3T_97* gene under the control of the P_spank_ promoter, adding 1 mM IPTG to ensure expression (see further information in ‘Plasmids and cloning’). We generated overlapping PCRs containing the erythromycin marker (including *lox* sites) and 1 kb of flanking region for the *phi3T_97* gene. After transformation of the PCRs into Δ6 phi3T strain, insertion of the erythromycin cassette was confirmed by PCR and sequencing, followed by removal of the antibiotic resistance cassette^25^. Briefly, plasmid pDR244 was transformed into strains harbouring the *lox*P-flanked antibiotic resistance cassette with selection for spectinomycin resistance at 30 °C to allow for Cre/*lox*-mediated loop-out of the cassette. Transformant colonies were streaked onto LB plates with 1 mM IPTG and incubated overnight at 42 °C for removal of the temperature-sensitive plasmid. Strains were screened for plasmid curing (loss of spectinomycin resistance) and the antibiotic resistance cassette (loss of erythromycin resistance). Clean mutants were confirmed by PCR and sequencing. The additional mutants used in this study listed in Supplementary Table 3 were generated by overlapping PCRs as described above without complementation in *trans*. To generate mutants in *maz*F, we used strain BKE04660 as a template for the PCR.

### Plasmids and cloning

All plasmids, primers and reagents are listed in Supplementary Tables 4, 5 and 6, respectively. Competent cell preparation and transformation was performed as follows: *B. subtilis* cells were grown in GM1 minimal media (SBase 1x (15 mM ammonium sulfate, 61 mM K_2_HPO_4_, 44 mM KH_2_PO_4_, 3.4 mM sodium citrate), 0.5% glucose, 0.1% yeast extract, 0.02% casein hydrolysate, 0.8 mM MgSO_4_, 0.025% d/l tryptophan, 0.02% l-methionine) to early stationary phase to induce natural competence. Then, 1 µg of DNA (PCR or plasmid) was added to a subculture of 500 μl cells grown in 5 ml GM2 (GM1 supplemented with 3.3 mM MgSO_4_ and 0.5 mM CaCl_2_), followed by incubation at 37 °C for 1 h with shaking at 210 r.p.m.^26^. Cultures were centrifuged at 6,000 *g* for 1 min, 800 μl of the supernatant removed, and the pellet resuspended and plated onto the relevant antibiotic plates. Plates were incubated at 37 °C overnight. For overexpression in *B. subtilis*, the genes were cloned into the *amy*E integration vector pDR110 under the control of the IPTG-inducible P_spank_ promoter^27^. The *phi3T_97* gene was cloned into a pDR110 where the spectinomycin resistance marker (*spec*) was replaced with a tetracycline (*tet*L) resistance marker. The pDR110 was digested with EcoRI and AsiSI restriction enzymes, and a 6,544-bp band was gel-extracted and ligated to a PCR amplifying the *tet*L resistance cassette from pDG1515. The reporter plasmid pDG1663 (ref. ^28^) was modified to introduce a new multicloning site with EcoRI-NotI-HindIII-SpeI-NheI-SphI-BamHI. Then, we cloned the P_spank_ promoter into the modified version of pDG1663, generating pDG1663+ P_spank_.

### β-galactosidase reporter assays

Strains were grown at 37 °C overnight in LB containing the appropriate antibiotics. Cultures were diluted 1:100 into fresh LB with 0.1 mM MnCl_2_ and 5 mM MgCl_2_, and grown at 37 °C with 210-r.p.m. shaking until an optical density (OD)_600_ of 0.2. When needed, 1 mM IPTG was added and cultures were grown for an additional hour before 1 ml of sample was taken and pelleted. OD_595_ values were measured for each sample. To measure β-galactosidase levels, the Miller method was used^29^. Briefly, cells were resuspended in Z buffer (60 mM Na_2_HPO_4_·7H_2_O, 40 mM NaH_2_PO_4_, 10 mM KCl, 1 mM MgSO_4_, 50 mM β-mercaptoethanol) and permeabilized with lysozyme. Following incubation with ONPG solution (4.0 mg ml^−1^ *o*-nitrophenyl-β-d-galactoside in Z buffer), the time taken for samples to change colour was noted and absorbance (A)_420_ readings were taken. Miller units were calculated as (1,000 × A_420_)/(reaction time (min) × OD_595_).

### Bacteriophage induction assay

For induction, overnight cultures were diluted 1:100 in LB with 0.1 mM MnCl_2_ and 5 mM MgCl_2_, and grown at 37 °C with 210-r.p.m. shaking until an OD_600_ of 0.2. This step was repeated twice to ensure the cells were in exponential growth. After the second growth, MC at 0.5 μg ml^−1^ was added. When experiments were performed to test the complementation of the mutants, 1 mM of IPTG was added simultaneously with MC. The induced cultures were incubated at 30 °C with 80-r.p.m. shaking for 4 h and then left overnight at room temperature. Following lysis, samples were filtered (0.2 μm) and lysates were stored at 4 °C until use.

### Total RNA extraction

An induction experiment was performed using wild-type phi3T, Δ*aim*R and Δ*aim*R evolved. After the second growth and before adding MC, 5 ml of sample was taken (time 0’), the rest of the culture was divided into two flasks with 10 ml of sample in each, adding MC (0.5 μg ml^−1^) to one flask. After 1 h incubation at 80 r.p.m. at 32 °C, 5 ml of each sample (time 60’ with or without MC treatment) were taken. For RNA extraction, each sample was mixed with two volumes of RNAprotect bacteria reagent (Qiagen) and incubated at room temperature for 5 min, then centrifuged. Pellets were snap frozen on dry ice and resuspended in 1 ml TRIzol reagent (Ambion) and lysed in a FastPrep-24 homogenizer (MP Biomedicals Lysing Matrix B tubes) using two cycles of 60 s at 6.5 m s^−2^ interrupted by 5 min incubation on ice. Total RNA was extracted using the TRIzol Plus RNA purification kit following manufacturer instructions. Genomic DNA was removed using an on-column DNase digestion step with an RNase-free DNase kit (Qiagen). Residual DNA was removed by a second DNase treatment using RQ1 DNase (Promega). Total RNA was measured using Nanodrop 2000/2000c (Thermo Scientific).

### Reverse transcription–PCR

The High-Capacity cDNA Reverse Transcription kit (Applied Biosystems) was used to convert RNA into complementary DNA. To verify the absence of genomic DNA in every RNA sample, the reverse transcription (RT) reaction was performed in the presence and absence of MultiScribe reverse transcriptase. In each reaction, 0.5 μg total RNA was subjected to RT following manufacturer instructions. The cDNA obtained was purified using QIAquick PCR purification kit (Qiagen) and 1 μl was used for PCR using Platinum *Taq* DNA polymerase HiFi (Life Technologies).

### Bacteriophage titring assay

Overnight cultures of the recipient strains (*B. subtilis ∆6* or with the corresponding integration vector) were diluted 1/100 in LB with 0.1 mM MnCl_2_ and 5 mM MgCl_2_, and then grown at 37 °C with 210-r.p.m. shaking until reaching an OD_600_ of 0.2. If needed, 1 mM IPTG was added. Then, 100 μl of recipient bacteria was infected with 100 μl of serial dilutions of phage lysate in phage buffer (PhB; 1 mM NaCl, 0.05 M Tris pH 7.8, 0.1 mM MnCl_2_, 5 mM MgCl_2_) at room temperature for 10 min. Then, 3 ml of phage top agar (LB with 0.1 mM MnCl_2_, 5 mM MgCl_2_ and 0.7% agar) at 55 °C was added to the culture–phage mix and immediately poured over phage base agar plates (LB with 0.1 mM MnCl_2_, 5 mM MgCl_2_ and 1.5% agar). Plaques were counted after overnight growth at 37 °C and photographed.

### Lysogenization assays

Lysogens were quantified by growing a recipient strain to OD_600_ 0.2. Lysates containing a kanamycin marker were serially diluted in PhB and 100 μl was added to 1 ml of the recipient bacteria in 12 ml tubes. After incubating at 37 °C for 30 min to allow the phage to infect bacteria, the mixture was then transferred to 1.5 ml Eppendorf tubes and centrifuged at 3,750 *g* for 1 min. The supernatant was removed and the bacterial pellet was resuspended in fresh LB broth before plating onto selective antibiotic LB agar plates. Plates were incubated overnight at 37 °C. The number of colony-forming units (c.f.u.) was calculated.

### Infection experiments

Cultures of the indicated strains were grown to OD_600_ 0.2 corresponding to ~3 × 10^7^ c.f.u. ml^−1^. If needed, 1 mM IPTG was added. Then, 15 ml of this culture was infected with the defined phage lysates at an MOI of 1:10 (phage:bacteria) and incubated at 30 °C and 80 r.p.m. At the indicated timepoints, samples were taken to assess the OD_600._ At the end timepoint, samples were taken to assess the number of lysogens and phage titres. For evaluating the phage titres, 1 ml of the co-culture was filtered (0.2 μm) to generate phage lysates. The number of phage particles was quantified by a titring assay against 168 Δ6. To quantify the number of lysogens, 1 ml of the co-culture was centrifuged at 3,750 *g* for 1 min and resuspended in 200 µl of fresh LB. The cells were then serially diluted in PBS and spotted onto LB kanamycin plates. All plates were incubated overnight at 37 °C and the number of plaque forming units (p.f.u.s) and c.f.u.s were calculated.

### Growth experiments

Overnight cultures of the relevant strains were diluted to OD_600_ 0.05 in LB with 0.1 mM MnCl_2_ and 5 mM MgCl_2_ and, if indicated, 1 mM IPTG, then grown at 37 °C with 120-r.p.m. shaking for 7 h. At the marked timepoints, samples were taken to assess the OD_600_.

### Statistical analysis

Statistical analyses were performed as indicated in the figure legends using GraphPad Prism 9 software. The *P* values represented in each figure are shown in the figure legends and detailed statistical test data, including exact *P* values, are provided in the source data file. In all figure descriptions, the number *n* represents biologically independent experiment results.

### Recombinant protein expression and purification

The *aim*X, *phi3T_93*, *phi3T_97* and *yos*L^*phi3T*^ genes were amplified using genomic DNA from *B. subtilis* strain 1L1. The *yosL*^*SPβ*^, *mazE* and *mazF* genes were amplified using genomic DNA from *B. subtilis* strain 168. In both cases, additional overhangs necessary for cloning were added (see primers in Supplementary Table 5). PCRs were cloned into the pLIC-SGC1 plasmid using NEBuilder HiFi DNA Assembly master mix (New England Biolabs). After sequence confirmation, plasmids were transformed into *E. coli* strain BL21 (DE3) RIL (Agilent) for protein overexpression.

For protein production, strains carrying the expression plasmid were grown overnight at 37 °C in 50 ml of LB with 50 µl ampicillin (100 μg ml^−1^) and 50 µl chloramphenicol (33 μg ml^−1^). The culture was used to inoculate 1 l of LB containing ampicillin and chloramphenicol, and was grown at 37 °C until OD_600_ 0.6–0.8. Then, the temperature was set to 20 °C and protein expression was induced with 0.4 mM IPTG. The culture was incubated at 20 °C for an additional 16 h. Cells were collected by centrifugation, washed with PBS and frozen at −20 °C until use.

For protein purification, cell pellets were resuspended in lysis buffer (50 mM Tris pH 8, 500 mM NaCl) and sonicated for 10 min on ice. Cell debris was separated by centrifugation at 10,000 *g* for 1 h. The supernatant was filtered (0.45 nm) and loaded onto a 1 ml TALON (Cytiva) affinity column equilibrated with buffer A (50 mM Tris pH 8, 250 mM NaCl), washed with 10 column volumes of buffer A supplemented with 25 mM imidazole and stepwise eluted with buffer B (buffer A with 500 mM imidazole). Fractions containing the purest protein as confirmed by SDS–PAGE were pooled and digested, if needed, with TEV protease (50:1 molar ratio protein:TEV) for 16 h at 4 °C while being dialysed against buffer A supplemented with 1 mM EDTA and 1 mM β-mercaptoethanol. Digested and dialysed samples were reloaded onto the TALON column pre-equilibrated with buffer A to remove the His-tag and the protease. The non-retained protein was concentrated by centrifugal filtration in a 3-kDa-cut-off Amicon Ultra system (Millipore) and loaded onto a Superdex 75 increase 10/300 (GE Healthcare) gel filtration column equilibrated in buffer A. After an isocratic elution with buffer A, the purest fractions were pooled, concentrated by centrifugal filtration, flash frozen in liquid nitrogen and stored at −80 °C until use. In all cases, protein purity levels were superior to 90% according to SDS–PAGE. AimX, phi3T_93, phi3T_97, YosL^phi3T^, YosL^SPβ^ and MazF^T78A^ yielded 5–15 mg of pure protein per litre of LB. MazE yielded ~2 mg of pure protein per litre of LB in a soluble and folded form as confirmed by circular dichroism (Supplementary Fig. 6).

### Protein crystallization

All crystals were obtained in the IBV-CSIC crystallogenesis facility at 21 °C using the sitting-drop vapour-diffusion method. His-phi3T_93 crystals were grown by mixing 0.3 µl protein solution at 10 mg ml^−1^ and 0.3 µl reservoir solution (0.1 M Tris pH 8, 5% glycerol and 0.6 M Na/K tartrate). phi3T_93 crystals were obtained by mixing 0.3 µl protein solution at 10 mg ml^−1^ and 0.3 µl reservoir solution (10% PEG 8000, 50 mM magnesium acetate and 100 mM sodium acetate). phi3T_93^L23D^ crystals were grown by mixing 0.3 µl protein solution at 10 mg ml^−1^ and 0.3 µl reservoir solution (18% w/v PEG 8000, 100 mM HEPES sodium salt pH 7.5, 200 mM calcium acetate). Crystals of AimX–phi3T_93 complex were obtained by co-crystallization. Samples were prepared by mixing 65 µl of phi3T_93 at 2 mM and 387 µl of AimX at 336 µM (1:1 molar ratio) and concentrating the mixture to 100 µl. Crystals were obtained by mixing 0.3 µl of the protein mixture and 0.3 µl reservoir solution (0.1 M HEPES pH 7.5, 10% PEG 8000 and 0.2 M calcium acetate).

### Data collection, structure solution and refinement

Before data collection, crystals were flash frozen in liquid nitrogen. For all phi3T_93 crystals, the corresponding crystallization solution was supplemented with 10% glycerol as cryoprotectant. AimX–phi3T_93 complex crystals were frozen in paratone. All diffraction experiments were carried out at 100 K.

His-phi3T_93, phi3T_93 and phi3T_93^L23D^ (PDB codes 8ANT, 8ANU and 8C8E, respectively) diffraction data were collected at Xaloc beamline, ALBA synchrotron in Barcelona using a wavelength of 0.97925 Å for phi3T_93 and 0.97918 Å for His-phi3T_93 and phi3T_93^L23D^. Data reduction was carried out in CCP4. Phases for His-phi3T_93 were obtained by ab initio phasing using ARCIMBOLDO LITE^30^ as implemented in CCP4i2 (ref. ^31^), whereas phi3T_93 and phi3T_93^L23D^ phases were obtained by molecular replacement using Phaser as implemented in Phenix^32^ and His-phi3T_93 or phi3T_93 as respective models. AimX–phi3T_93 (PDB code 8ANV) crystals were collected at beamline I04 of Diamond Light Source synchrotron (Oxfordshire, UK) using a wavelength of 0.9795 Å. Data reduction was carried out by the beamline software pipeline. The structure of the complex was solved at 2.2 Å resolution by molecular replacement using the phi3T_93 structure as a model for Phaser^33^ in CCP4 suite. Following phase determination, model refinement was carried out combining manual building with Coot^34^ (v.0.9.8.8) and computational refinement using phenix.refine^35^. Extended Data Table 2 details all the X-ray data collection and refinement statistics.

### Structure prediction

The predicted structures of YosL from phi3T and SPβ phages were determined with AlphaFold2 run online in Google ColabFold v.1.5.2 using default (auto) settings^36^. Proteins with structural similarity to the structures determined experimentally or predicted by Alphafold were searched using the DALI server^37^. Structures were visualized and figures prepared with PyMOL 2.1 (Schrödinger) and Chimera (https://www.cgl.ucsf.edu/chimera/).

### Binding kinetics analysis

Binding kinetics were measured by biolayer interferometry using the BLiTZ system (FortéBio, PALL) and Ni-NTA biosensors (Sartorius) at room temperature. All proteins were diluted in binding buffer (25 mM Tris pH 8, 150 mM NaCl, 3 mM imidazole and 0.005% Tween 20). Measurements were carried out by immobilizing the protein bait at 0.3 mg ml^−1^ for 120 s to the biosensors through its N-terminal His-tag. Association and dissociation were monitored for 60 s each using the protein prey at concentrations from 1.25 to 0.07813 µM, obtained by 1:2 serial dilution from the highest concentration. Data analysis was performed using BLiTZ pro software (ForteBio).

### SEC–MALS experiments

For SEC–MALS assays, 20 µl of phi3T_93 (wt and the different mutants) or AimX constructs at 1 mg ml^−1^ were injected onto a Shodex KW-402.5-4F column, equilibrated with 100 mM HEPES pH 7.5 and 250 mM NaCl using a Shimadzu HPLC equipped with a manual injector at a flow rate of 0.3 ml min^−1^. MALS analysis was performed using a miniDAWN Treos MALS detector followed by an optilab T-rEX and a DLS detector (Wyatt). Data analysis was performed using Astra software (Wyatt).

### Circular dichroism

Data collection was performed between wavelengths 200–275 nm in a J-1500 spectrometer with quartz cuvettes, using 150 µl 27 µM MazE diluted in PBS (20 mM) at pH 7.0. Millidegrees were used for secondary structure determination. The composition of secondary structures was estimated from the data using the software BeStSel^38^.

### Native-PAGE

A modified version of Native-PAGE analysis^39^ was used to study the dynamics of complex formation. Briefly, a MazE–MazF complex was initially obtained by mixing MazE (30 µM) and MazF (60 μM) (ratio 1:2) in buffer A (200 mM NaCl, 50 mM Tris-HCl pH 8.0) and incubating at room temperature for 15 min. After complex formation, AimX was added to the MazE–MazF mixture to a final concentration of 30 µM at 20 min, 10 min and 5 min before loading the sample into the gel. The samples were separated in 8% acrylamide native gel (100 V, 4 °C, 2 h) and visualized by Coomassie blue staining.

### Reporting summary

Further information on research design is available in the Nature Portfolio Reporting Summary linked to this article.

### Supplementary information

Supplementary Information Supplementary Figs. 1–5, Tables 1–6 and references for the supplementary information.

Reporting Summary

Supplementary Data Source data for Supplementary Figs. 5 and 6.

### Source data

Source Data **Fig. 1** Source data. Source Data **Fig. 2** Source data. Source Data **Fig. 3** Source data. Source Data **Fig. 4** Source Data **Fig. 5** Source Data **Extended Data Fig. 1** Source Data **Extended Data Fig. 2** Source data. Source Data **Extended Data Fig. 3** Source data. Source Data **Extended Data Fig. 4** Source data. Source Data **Extended Data Fig. 5** Source data.

## Figures and Tables

**Extended Data Table 1:**
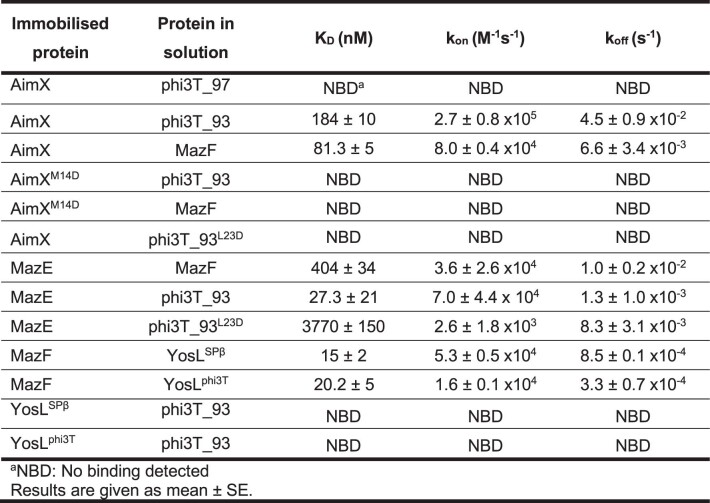
BLI calculated binding affinities

**Extended Data Table 2:**
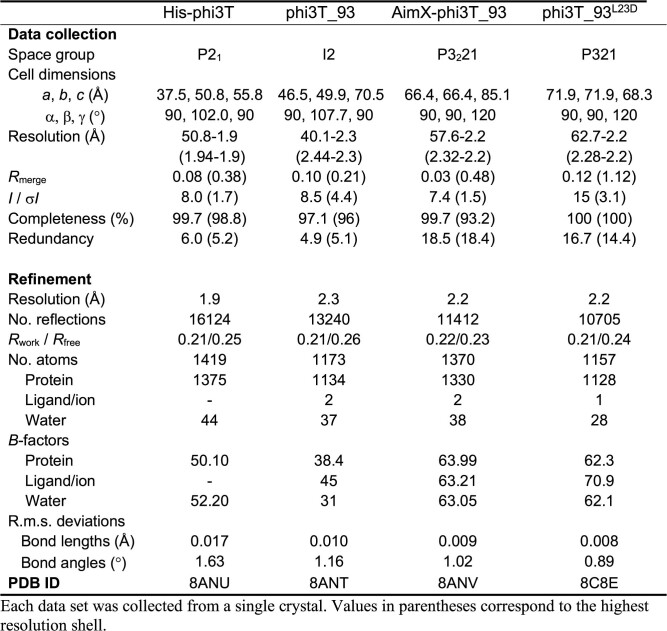
Data collection and refinement statistics

## Data Availability

The atomic coordinates of the phi3T_93, His-phi3T_93, phi3T_93^L23D^ and AimX-phi3T_93 complex have been deposited at http://www.pdb.org, with PDB codes 8ANT, 8ANU, 8C8E and 8ANV, respectively. The previously determined structures used in this study are available from the PDB (http://www.pdb.org) under the accession codes indicated. The rest of the data are available in the main text, supplementary materials and auxiliary files. Plasmids and bacterial strains generated during this work are listed in Supplementary Tables 3 and 4 and are available upon request.  Source data are provided with this paper.
