# Supplementary material for: Antagonistic interactions between phage and host factors control arbitrium lysis–lysogeny decision
Source: Nat Microbiol. 2024 Jan 4;9(1):161–72. doi: 10.1038/s41564-023-01550-4 (PMC10769878; doi:10.1038/s41564-023-01550-4)
Supplement: Supplementary file 1 — Supplementary Figs. 1–5, Tables 1–6 and references for the supplementary information. [file 41564_2023_1550_MOESM1_ESM.pdf]

nature microbiology

Article https://doi.org/10.1038/s41564-023-01550-4

Antagonistic interactions between phage and host factors control arbitrium lysis–lysogeny decision

In the format provided by the authors and unedited

CONTENTS

**Supplementary Figures**	page
Supplementary Figure 1	2
Supplementary Figure 2	3
Supplementary Figure 3	4
Supplementary Figure 4	5
Supplementary Figure 5	6
Supplementary Figure 6	7
**Supplementary Tables**	
Supplementary Table 1	8
Supplementary Table 2	9
Supplementary Table 3	10
Supplementary Table 4	13
Supplementary Table 5	15
Supplementary Table 6	20
**References**	22

Supplementary Figures

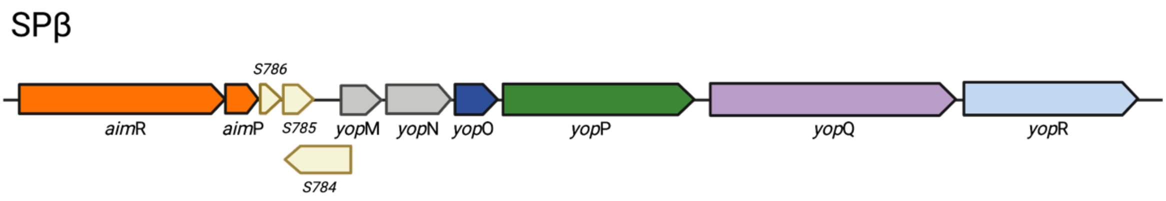

Supplementary Figure 1. Genetic organisation of the phi3T and SPβ arbitrium systems and their related operons. Genetic organisation of the arbitrium genes, aimR and aimP, followed by the operon directly downstream. Colours denote putative functions according to BLASTP and previously published results; orange, arbitrium genes; light yellow, sRNAs; grey, unknown function; navy blue, HTH_XRE domain; green, integrase domain; purple, ParB domain; light blue, phage repressor. Shown was created with Biorender.com

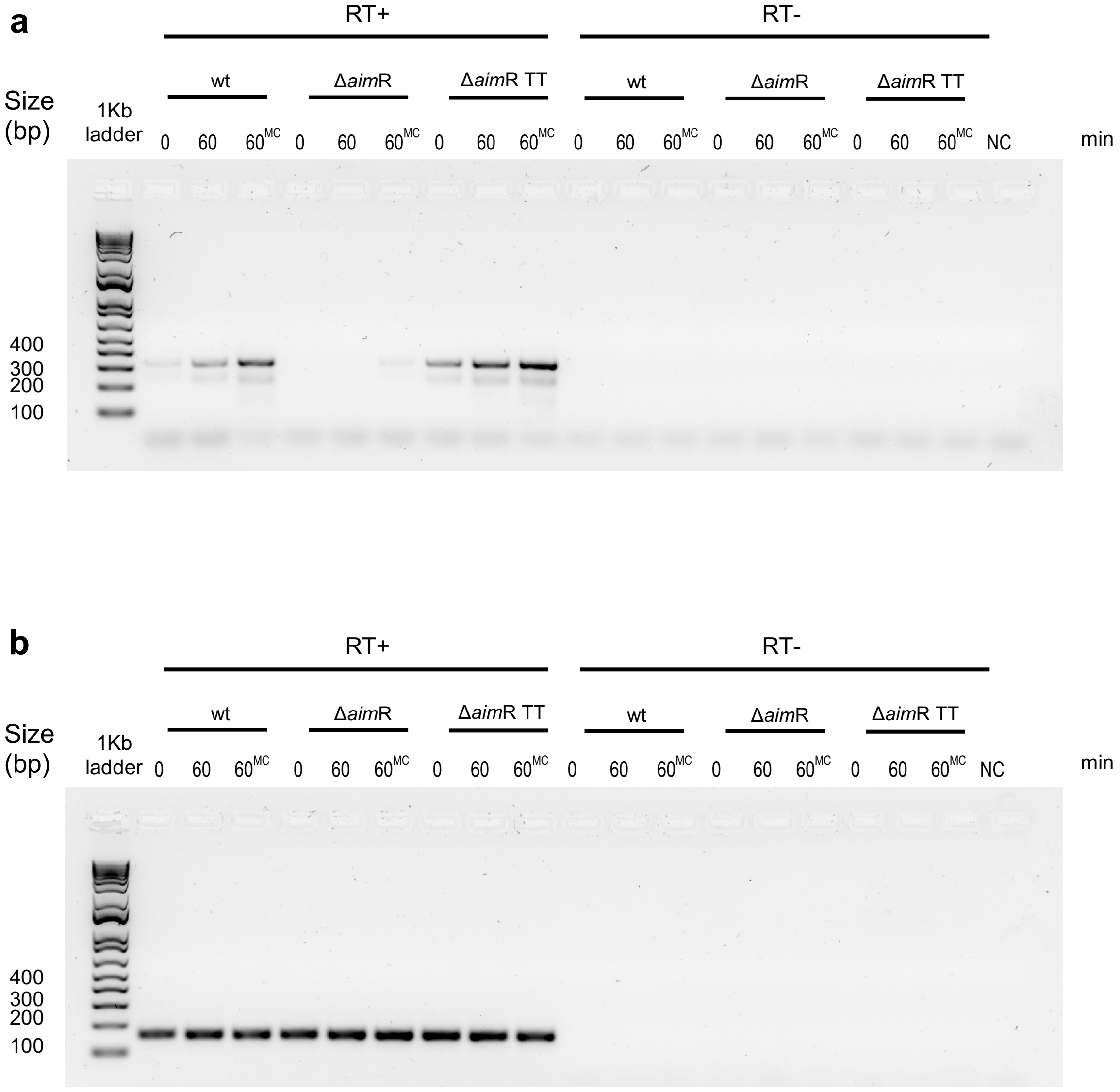

Supplementary Figure 2. Agarose gel electrophoresis of RT-PCR products. a, RT-PCR products for primers amplifying the region encompassing the *aim*P and *aim*X genes. **b,** RT-PCR products for primers amplifying the *B. subtilis gyr*B gene. One total RNA extraction was performed from cultures of Δ6 lysogenic for phages phi3T wt, Δ*aim*R and ⊗*aim*R evolved (⊗*aim*R TT) grown at different conditions: times 0, 60 min and 60 min after MC induction (60^{MC}). Reverse transcription reaction (RT) was performed for each sample to synthesise cDNA. To verify the absence of genomic DNA in the RNA samples, the RT was performed in the presence (RT+) and absence (RT-) of the MultiScribe reverse transcriptase. The products of the RT reactions were used as templates for one PCR reaction and 5 µl of each PCR product was loaded in a 1.5 % agarose gel. 1Kb ladder and sizes (bp) indicated. NC: PCR negative control.

a

```
aimX    ATGAAAAGAGCATTAGGTAAAGCAATATCTTATGAAGAAATGGCAAAAGGGTACGA
aimXnt  ATGAAGAGGGCGCTGGGGAAGGCGATCAGCTACGAGGAGATGGCCAAGGGTTATGA

aimX    GGAAATGGCTGCAATCAATTCAATAATTGCTCAAGAGGACAACCATCTTGAGAATG
aimXnt  AGAGATGGCGGCCATAAACAGTATCATAGCGCAGGAAGATAATCACTTGGAAAACG

aimX    AAGCGGAAATGATTAAAACAAGGTATAAAACCCTGGCTTCATGA
aimXnt  AGGCAGAGATGATCAAGACGCGGTACAAGACATTAGCGAGTTGA
```

b

```
AimX    MKRALGKAISYEEMAKGYEEMAAINSIIAQEDNHLENEAEMIKTRYKTLAS*
AimXnt  MKRALGKAISYEEMAKGYEEMAAINSIIAQEDNHLENEAEMIKTRYKTLAS*
```

c

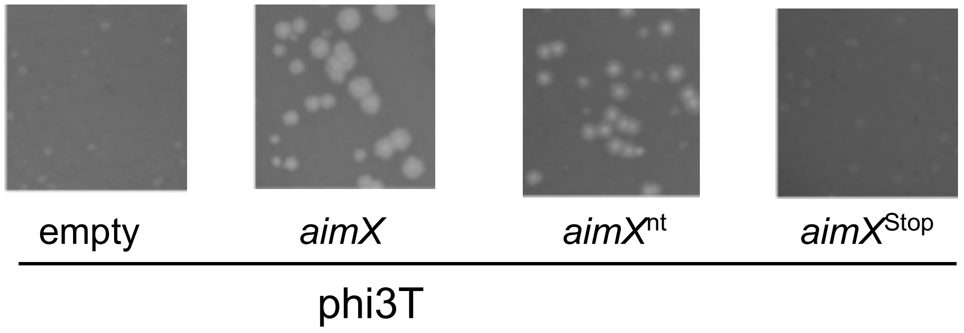

Supplementary Figure 3. Nucleotide and protein sequence comparison between *aim*X and *aim*X variant. An *aim*X variant was generated by modifying the nucleotide sequence without changing the encoded amino acid sequence. **a,** Nucleotide sequence alignment between *aim*X and the *aim*X variant. The upper line shows the wt *aim*X DNA sequence; the lower line shows the *aim*X variant DNA sequence. The conserved nucleotides are highlighted in blue. **b**, Protein sequence alignment between wt AimX and the AimX variant. The upper line shows the AimX protein sequence; the lower line shows the aimX variant protein sequence. The conserved amino acids are highlighted in blue. **c**, Complementation of the phi3T Δ*aim*R mutant in recipient. Strains lysogenic for phages phi3T Δ*aim*R were MC induced. The morphology of plaques following phi3T Δ*aim*R infection of strains 168 Δ6 expressing different versions of the *aim*X gene were photographed.

a

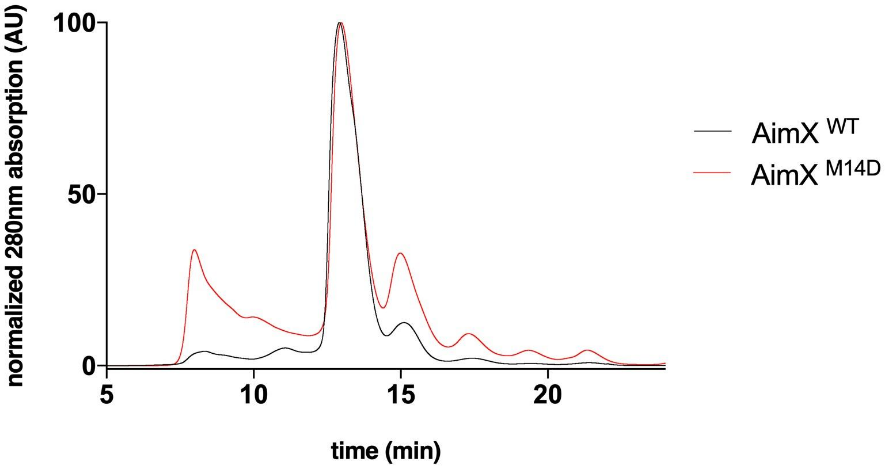

b

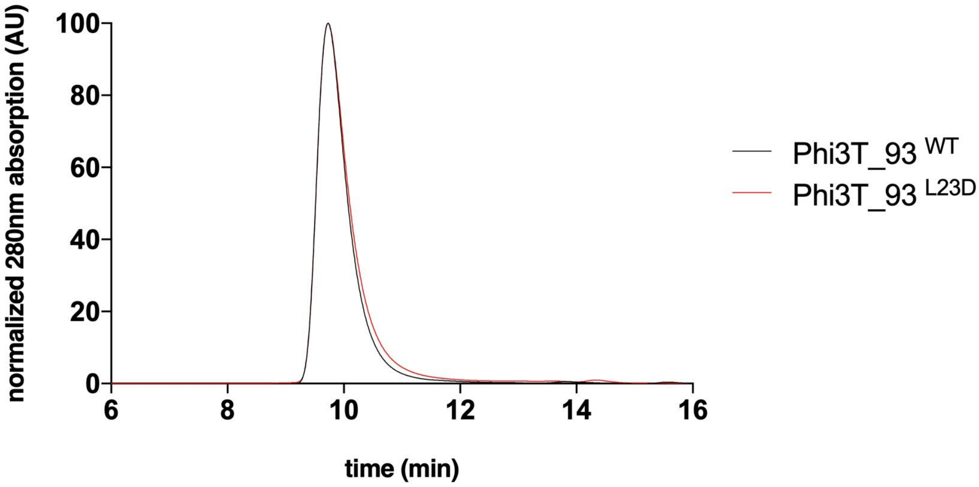

c

	Molecular Weight (kDa)	
**Protein**	**Theoretical**	**Calculated**
His-AimX ^{WT}	8,4	9,9 ± 0,47
phi3T_93	10,9	23,5 ± 1,92
phi3T_93 ^{L23D}	10,9	19,9 ± 1,01

Supplementary Figure 4. Point mutations on AimX and phi3T_93 phage do not affect protein behaviour in solution. a, Size exclusion chromatography pattern for AimX ^{WT} and AimX ^{M14D} (His-tagged) in a Superdex 75 increase column. **b,** Size exclusion chromatography pattern for phi3T_93, and phi3T_93 ^{L23D} in a Shodex KW-402.5-4F column. **c,** Molecular weight in kDa for AimX, phi3T_93, phi3T_93 ^{L23D} and as calculated using SEC-MALS.

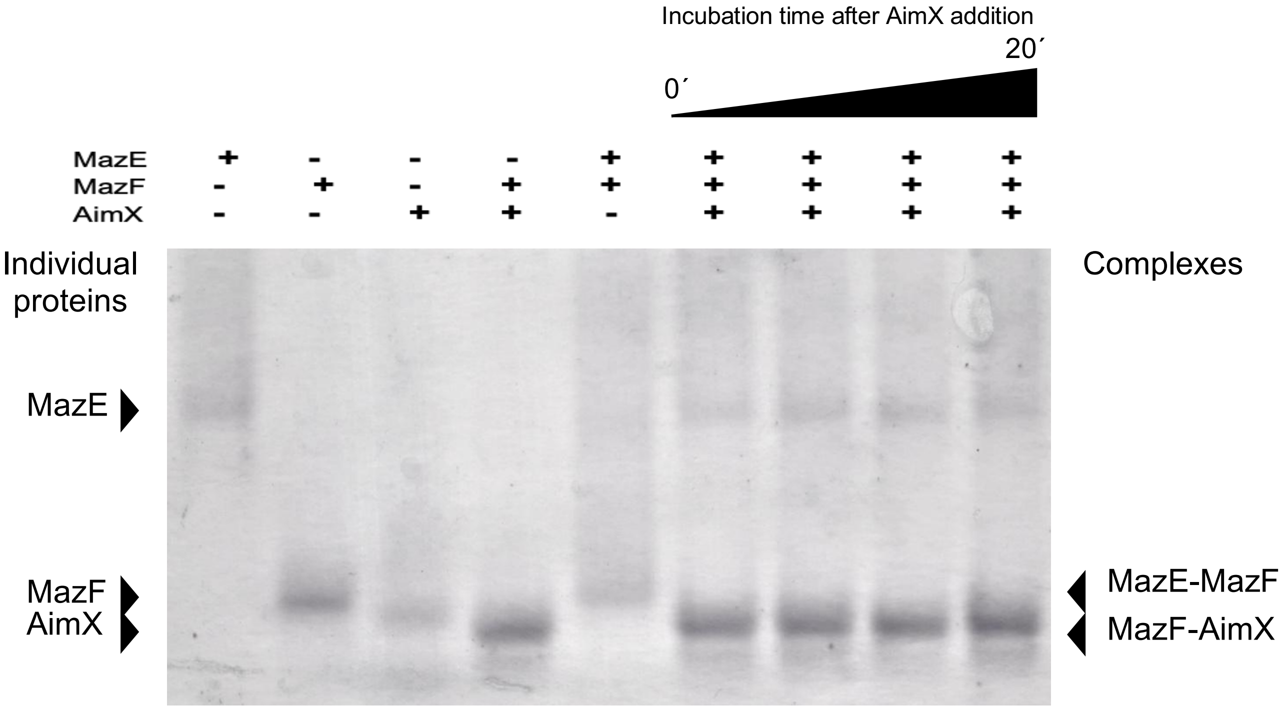

Supplementary Figure 5. Competition assays by native gel assay. Native gel mobility shift assays tested the binding preference of MazF between its partner antitoxin MazE and AimX. The pre-formed MazE-MazF complex was mixed with equimolar amounts of AimX and the formation of the MazE-MazF and AimX-MazF complexes at different times (from 0 to 20 minutes) were visualised by native gels showing the rapid disappearance of the MazE-MazF complex by the formation of the AimX-MazF complex. A representative of five gels is shown. Source Data Supplementary Information

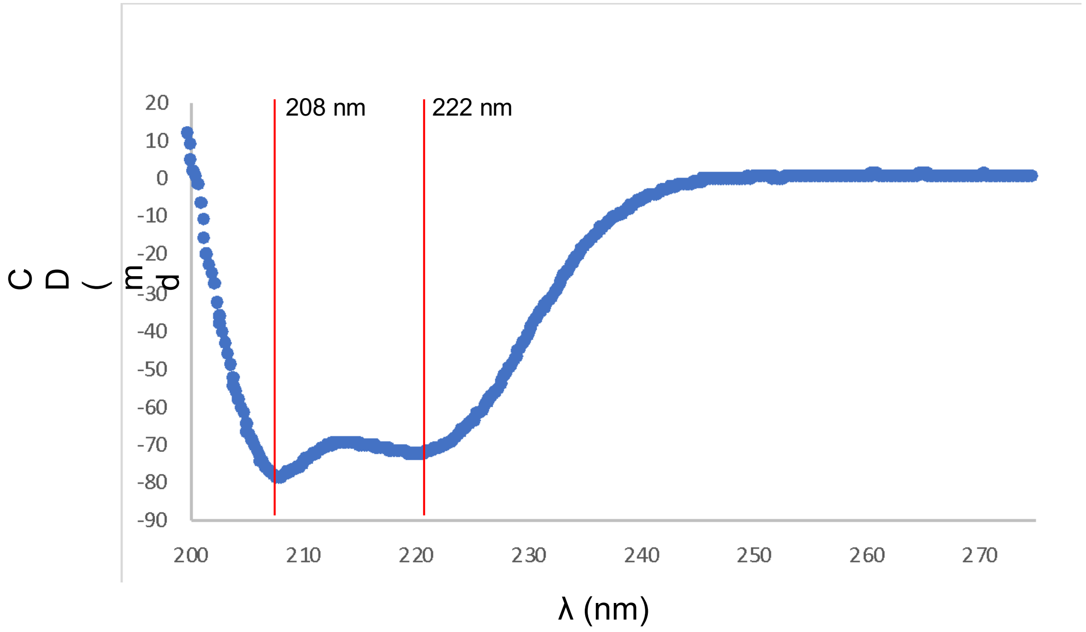

Secondary structure	Calculated CD	Crystal structure
Helix	74,0 %	79,5 %
Turn	8,6 %	3,8 %
Coil	17,4 %	16,7 %

Supplementary Figure 6. Circular dichroism (CD) spectrum of recombinant MazE. The CD spectrum of recombinant MazE at pH 7.0 showed two negative peaks at 208 and 222 nm (*upper panel*) that after curve fitting with BeStSel software indicates a high content of alpha helix (lower panel) in agreement with the secondary structures of MazE determined by the DSSP form its crystal structure (PDM 4ME7). Source Data Supplementary Information

Supplementary Tables

Supplementary Table S1. Mutations identified in evolved ΔaimR phages.

Strain	phage	DNA region	nt position (phi3T KY030782)	nt position (SPβ NC_001884)	Mutation
JP23123	phi3T	TT between *aim*P and *aim*X	70222		G-C
JP23124	phi3T	TT between *aim*P and *aim*X	70222		G-C
JP23126	phi3T	TT between *aim*P and *aim*X	70222		G-C
JP23127	phi3T	TT between *aim*P and *aim*X	70222		G-C
JP23130	phi3T	TT between *aim*P and *aim*X	70222		G-C
JP23131	phi3T	TT between *aim*P and *aim*X	70222		G-C
JP23775	SPβ	TT between *aim*P and *aim*X		77686	G-T
JP23776	SPβ	Intergenic region *yok*L-*yol*A		12942	A-G
	SPβ	TT between *aim*P and *aim*X		77686	G-T
JP23777	SPβ	TT between *aim*P and *aim*X		77686	G-T

Supplementary Table S2. Interactions of phi3T_93 (dimer) with AimX and Histag in AimX-phi3T_93 and His- phi3T_93 structures, respectively.

phi3T_93		AimX		Linker-Histag	
**Chain**	**Residue**	**Chain**	**Residue**	**Chain**	**Residue**
A	K5	C	E20		
A	Y6	C	E13		
A		C	K16		
A		C	G17		
A		C	E20		
A	Y7	C	E20		
A	W48			A	F-2
A	F51	C	Y18		
B	K13	C	M21		
B	W16	C	M14		
B	W16	C	G17		
B	W16			A	M1
B	K17	C	Y18		
B	H19	C	M14		
B	M20	C	Y11	A	F-2
B		C	M14	A	M1
B		C	A15		
B	L23	C	M14		
B	K24			A	F-2
B	E27			A	V-11
B				A	N-5
B	G61			A	Y-3
B	K62			A	Y-3
B	P63			A	Y-3
B	V64	C	M14		
B	I65	C	E13	A	S0
B				A	M1
B	V67			A	S0

Supplementary Table S3. Strains used in this study

Strain	Genotype or description	Reference or source
Bacillus subtilis		
168 (1A700) (JP13763)	*trpC2*	[1]; BGSC
Δ6 (1A1299) (JP19666)	*trpC2*; ΔSPβ ; subclacin 168-sensitive; Δ*skin*; ΔPBSX; Δprophage 1; Δ*pks*::Cm; Δprophage 3; Cm^r	[2]; BGSC
BKE04660 (JP23767)	*trp*C2 Δ*maz*F::erm	[3];BGSC
JP21870	Δ6 lysogenic phi3T *phi3T_5*::*kan*	[4]
JP23227	Δ6 lysogenic phi3T *phi3T_5*::*kan* Δ*phi3T_93*	This study
JP23801	Δ6 lysogenic phi3T *phi3T_5*::*kan*; *amy*E::P_{spank} *phi3T_97*	This study
JP23811	Δ6 lysogenic phi3T *phi3T_5*::*kan* Δ*phi3T_97; amy*E::P_{spank} *phi3T_97*	This study
JP19679	Δ6 *amy*E::P_{spank}	[4]
JP23230	Δ6 *amy*E::P_{spank} *phi3T_93*	This study
JP23800	Δ6 *amy*E::P_{spank} *phi3T_97*	This study
JP22453	Δ6 lysogenic phi3T *phi3T_5*::*kan* Δ*aim*R	[4]
JP23123	Δ6 lysogenic phi3T *phi3T_5*::*kan* Δ*aim*R E1	This study
JP23124	Δ6 lysogenic phi3T *phi3T_5*::*kan* Δ*aim*R E2	This study
JP23126	Δ6 lysogenic phi3T *phi3T_5*::*kan* Δ*aim*R E3	This study
JP23127	Δ6 lysogenic phi3T *phi3T_5*::*kan* Δ*aim*R E4	This study
JP23130	Δ6 lysogenic phi3T *phi3T_5*::*kan* Δ*aim*R E5	This study
JP23131	Δ6 lysogenic phi3T *phi3T_5*::*kan* Δ*aim*R E6	This study
JP22949	Δ6 lysogenic SPβ *yok*I::*kan* ΔaimR	[4]
JP23775	Δ6 lysogenic SPβ *yok*I::*kan* ΔaimR E1	This study
JP23776	Δ6 lysogenic SPβ *yok*I::*kan* ΔaimR E2	This study
JP23777	Δ6 lysogenic SPβ *yok*I::*kan* ΔaimR E3	This study
JP23407	Δ6 *thr*C::P_{spank}	This study
JP23408	Δ6 *thr*C::P_{spank} - transcriptional terminator upstream *aim*X (TT) - *lacZ*	This study
JP23409	Δ6 *thr*C::P_{spank} - transcriptional terminator upstream *aim*X mutated version (TT*) - *lacZ*	This study
JP23748	Δ6 *amy*E::P_{spank} ; *thr*C::*aim*P - *aim*X phi3T - *lacZ*	This study
JP23728	Δ6 *amy*E::P_{spank} ; *thr*C::P*aim*P - *aim*P - *aim*X phi3T - *lacZ*	This study
JP23750	Δ6 *amy*E::P_{spank} ; *thr*C::P*aim*P - *aim*P - *aim*X phi3T transcriptional terminator mutated (TT*) - *lacZ*	This study
JP23751	Δ6 *amy*E::P_{spank} ; *thr*C::P*aim*P - *aim*P - *aim*X phi3T mutant in AimR binding site - *lacZ*	This study
JP23752	Δ6 *amy*E::P_{spank} ; *thr*C::P*aim*P - *aim*P - *aim*X phi3T increased distance - *lacZ*	This study
JP23753	Δ6 *amy*E::P_{spank} ; *thr*C::P*aim*P - *aim*P - *aim*X phi3T decreased distance - *lacZ*	This study
JP23755	Δ6 *amy*E::P_{spank} $aimR_{3T}$; *thr*C::*aim*P - *aim*X phi3T - *lacZ*	This study
JP23730	Δ6 *amy*E::P_{spank} $aimR_{3T}$; *thr*C::P*aim*P - *aim*P - *aim*X phi3T - *lacZ*	This study

Supplementary Table S3. Strains used in this study (continuation)

Strain	Genotype or description	Reference or source
JP23757	Δ6 *amy*E::P_{spank} *aim*R_{3T} ; *thr*C::P*aim*P - *aim*P - *aim*X phi3T transcriptional terminator mutated (TT*) - *lac*Z	This study
JP23758	Δ6 *amy*E::P_{spank} *aim*R_{3T} ; *thr*C::P*aim*P - *aim*P - *aim*X phi3T mutant in AimR binding site - *lac*Z	This study
JP23759	Δ6 *amy*E::P_{spank} *aim*R_{3T} ; *thr*C::P*aim*P - *aim*P - *aim*X phi3T increased distance - *lac*Z	This study
JP23760	Δ6 *amy*E::P_{spank} *aim*R_{3T} ; *thr*C::P*aim*P - *aim*P - *aim*X phi3T decreased distance - *lac*Z	This study
JP23779	Δ6 *amy*E::P_{spank} ; *thr*C::P_{spank} - *S784* - *S785* SPβ - *lac*Z	This study
JP23789	Δ6 *amy*E::P_{spank} ; *thr*C::P_{spank} - *S784* - *S785* SPβ transcriptional terminator mutated (TT*) - *lac*Z	This study
JP23780	Δ6 *amy*E::P_{spank} aim$R_{SP\beta}$; *thr*C::P_{spank} - *S784* - *S785* SPβ - *lac*Z	This study
JP23790	Δ6 *amy*E::P_{spank} aim$R_{SP\beta}$; *thr*C::P_{spank} - *S784* - *S785* SPβ transcriptional terminator mutated (TT*) - *lac*Z	This study
JP23806	Δ6 *amy*E::P_{spank} ; *thr*C::P*aim*P - *aim*P - *aim*X phi3T binding site Aim$R_{SP\beta}$ - *lac*Z	This study
JP23807	Δ6 *amy*E::P_{spank} *aim*R_{3T} ; *thr*C::P*aim*P - *aim*P - *aim*X phi3T binding site Aim$R_{SP\beta}$ - *lac*Z	This study
JP23808	Δ6 *amy*E::P_{spank} *aim*$R_{SP\beta}$; *thr*C::P*aim*P - *aim*P - *aim*X phi3T binding site Aim$R_{SP\beta}$ - *lac*Z	This study
JP22518	Δ6 lysogenic phi3T *phi3T_5*::*kan* Δ*aim*R; *amy*E::P_{spank}	[4]
JP22519	Δ6 lysogenic phi3T *phi3T_5*::*kan* Δ*aim*R; *amy*E::P_{spank} *aim*R_{3T}	[4]
JP23743	Δ6 lysogenic phi3T *phi3T_5*::*kan* Δ*aim*R; *amy*E::P_{spank} *aim*X_{3T}	This study
JP23744	Δ6 lysogenic phi3T *phi3T_5*::*kan* Δ*aim*R; *amy*E::P_{spank} *aim*$X_{3T\ nucleotide\ variant}$	This study
JP23745	Δ6 lysogenic phi3T *phi3T_5*::*kan* Δ*aim*R; *amy*E::P_{spank} *aim*X_{3T} L5*	This study
JP23746	Δ6 lysogenic phi3T *phi3T_5*::*kan* Δ*aim*R; *amy*E::P_{spank} *aim*X_{3T} M14D	This study
JP22775	Δ6 lysogenic phi3T *phi3T_5*::*kan* Δ*aim*X	This study
JP24244	Δ6 lysogenic phi3T *phi3T_5*::*kan* Δ*aim*R; *amy*E::P_{spank} *yos*L_{3T}	This study
JP24213	Δ6 lysogenic phi3T *phi3T_5*::*kan* Δ*yos*L	This study
JP24214	Δ6 lysogenic phi3T *phi3T_5*::*kan* Δ*yos*L Δ*aim*X	This study
JP24218	Δ6 lysogenic phi3T *phi3T_5*::*kan* Δ*yos*L Δ*aimX; amy*E::P_{spank}	This study
JP24217	Δ6 lysogenic phi3T *phi3T_5*::*kan* Δ*yos*L Δ*aimX; amy*E::P_{spank} *aim*X_{3T}	This study
JP24216	Δ6 lysogenic phi3T *phi3T_5*::*kan* Δ*yos*L Δ*aimX; amy*E::P_{spank} *yos*L_{3T}	This study
JP22789	Δ6 *amy*E::P_{spank} *aim*X_{3T}	This study
JP24219	Δ6 *amy*E::P_{spank} *yos*L_{3T}	This study
JP24211	Δ6 Δ*maz*F	This study
JP24212	Δ6 Δ*maz*EF	This study
JP24220	Δ6 Δ*maz*F *amy*E::P_{spank}	This study
JP24221	Δ6 Δ*maz*F *amy*E::P_{spank} *maz*F	This study
JP24222	Δ6 Δ*maz*F *amy*E::P_{spank} *maz*F T48A	This study
JP24223	Δ6 Δ*maz*EF *amy*E::P_{spank}	This study

Supplementary Table S3. Strains used in this study (continuation).

Strain	Genotype or description	Reference or source
JP24226	Δ6 Δ*maz*EF *amy*E::P_{spank} *maz*F	This study
JP24229	Δ6 Δ*maz*EF *amy*E::P_{spank} *maz*F; lysogenic phi3T *phi3T_5*::*kan*	This study
JP24230	Δ6 Δ*maz*EF *amy*E::P_{spank} *maz*F; lysogenic phi3T *phi3T_5*::*kan* Δ*aim*R E1	This study
JP24228	Δ6 Δ*maz*F; lysogenic phi3T *phi3T_5*::*kan* Δ*yos*L Δ*aim*X	This study
JP23771	Δ6 *amy*E::P_{spank} *phi3T_93* L23D	This study
Escherichia coli		
BL21-CodonPlus (DE3)-RIL	E. coli B F– ompT hsdS(r – m –) dcm+ Tetr gal λ(DE3) endA Hte [argU ileY BB	Agilent

Supplementary Table S4. Plasmids used in this study

Plasmid	Description	Reference or source
pDR244	*B. subtilis* thermosensitive vector containing Cre recombinase that allows excision of DNA fragments flanked by *lox*P sites	[3]
pDR110	*B. subtilis amy*E integration vector containing IPTG-inducible P_{spank} promoter	[5]
pDG1515	*E. coli* BluscriptKS+ plasmid source for tetracline resistance marker (*tet*L)	[6]
pDR110-*tet*L	*B. subtilis amy*E integration vector containing IPTG-inducible P_{spank} promoter with *tet*L	This study
pJP2827	*phi3T_93* gene cloned in integration vector pDR110	This study
pJP2826	*phi3T_97* gene cloned in integration vector pDR110-*tet*L	This study
pJP2965	*phi3T_97* gene cloned in integration vector pDR110	This study
pJP2340	*aim*$R_{SP\beta}$ gene cloned in integration vector pDR110	[4]
pJP2801	*aim*R_{3T} gene cloned in integration vector pDR110	[4]
pDG1663	*B. subtilis* reporter vector containing β-gal gene, integrates at *thr*C	[7]
pDG1663+	*B. subtilis* reporter vector containing β-gal gene, integrates at *thr*C. Modified MCS (EcoRI-NotI-HindIII-SpeI-NheI-SphI-BamHI)	This study
pDG1663+ P_{spank}	*B. subtilis* reporter vector containing β-gal gene, integrates at *thr*C, containing IPTG-inducible P_{spank} promoter	This study
pJP2808	pDG1663+ P_{spank} - transcriptional terminator upstream *aim*X phi3T (TT)	This study
pJP2809	pDG1663+ P_{spank} - transcriptional terminator upstream *aim*X phi3T mutated version (G-C) (TT*)	This study
pJP2810	pDG1663+ *aim*P - *aim*X phi3T (No PR*aim*P)	This study
pJP2811	pDG1663+ P*aim*P - *aim*P - *aim*X phi3T (wt)	This study
pJP2812	pDG1663+ P*aim*P - *aim*P - *aim*X phi3T transcriptional terminator mutated version (G-C) (TT*)	This study
pJP2813	pDG1663+ P*aim*P - *aim*P - *aim*X phi3T mutant AimR binding site (ΔBS)	This study
pJP2814	pDG1663+ P*aim*P - *aim*P - *aim*X phi3T increased distance (Incr)	This study
pJP2815	pDG1663+ P*aim*P - *aim*P - *aim*X phi3T decreased distance (Decr)	This study
pJP2816	pDG1663+ P_{spank} - *S784* - *S785* SPβ (wt)	This study
pJP2817	pDG1663+ P_{spank} - *S784* - *S785* SPβ transcriptional terminator mutated version (G-T) (TT*)	This study
pJP2821	pDG1663+ P*aim*P - *aim*P - *aim*X phi3T AimR binding site SPβ	This study
pJP2822	*aim*X_{3T} gene cloned in integration vector pDR110	This study
pJP2823	*aim*X_{3T} nucleotide variant gene cloned in integration vector pDR110	This study
pJP2824	*aim*X_{3T} L5* gene cloned in integration vector pDR110	This study
pJP2825	*aim*X_{3T} M14D gene cloned in integration vector pDR110	This study
pJP3004	*yos*L_{3T} gene cloned in integration vector pDR110	This study

Supplementary Table S4. Plasmids used in this study (continuation)

Plasmid	Description	Reference or source
pJP3005	*maz*F gene cloned in integration vector pDR110	This study
pJP3006	*maz*F T48A gene cloned in integration vector pDR110	This study
pJP2829	*phi3T_93* L23D gene cloned in integration vector pDR110	This study
pLIC-AimX	phi3T *aimX* gene with N-terminal His-tag cloned into pLIC SGC1	This study
pLIC-AimXM14D	phi3T *aimX*^{M14D} gene mutant with N-terminal His-tag cloned into pLIC SGC1	This study
pLIC- phi3T_97	*phi3T_97* gene with N-terminal His-tag cloned into pLIC SGC1	This study
pLIC- phi3T_93	*phi3T_93* gene with N-terminal His-tag cloned into pLIC SGC1	This study
pLIC-phi3TL23D	*phi3T_93* L23D gene with N-terminal His-tag cloned into pLIC SGC1	This study
pLIC-MazF	*B. subtilis mazF* gene with N-terminal His-tag cloned into pLIC SGC1	This study
pLIC-MazFT48A	*B. subtilis mazF* T48A gene mutant N-terminal His-tag cloned into pLIC SGC1	This study
pLIC-MazE	*B. subtilis mazE* gene with N-terminal His-tag cloned into pLIC SGC1	This study

Supplementary Table S5. Primers used in this study

Mutants	Oligonucleotides		Sequence (5’-3’)
erm marker with *lox*P	ErmR-1m		GCAGGCGAGAAAGGAGAGAGAACGCAAGGAGAGGCACGCGAGGGAGGAAAGGCAGGATACCGTTCGTATAGCATACATTATACGAAGTTATGAATTC
	ErmR-2c		CGAGGCTCCTGTCACTGCTTCGCTCTGCTTCGGTGTCGTCGCCGTATCTGTGCTCTCTCTACCGTTCGTATAATGTATGCTATACGAAGTTATCTCGAG
phi3T_93::*erm*	Forward Flanking	phi3T_93-1F	GCCATTACTTCAACTCGAACGAATG
		phi3T_93-2R	GTTCTCTCTCCTTTCTCGCCTGCTTTATTTTTATTCTCCTTATATGCATTTAATTGTG
	Reverse Flanking	phi3T_93-3F	GCGAAGCAGTGACAGGAGCCTCGCAAGTATATACTTAAAGTAGGTGTTTTATTG
		phi3T_93-4R	CAGTTCATGCTCACCATACTCATTC
phi3T_97::*erm*	Forward Flanking	phi3T_97-1F	CTATAATACTCAACGGGAAGGCAG
		phi3T_97-2R	GTTCTCTCTCCTTTCTCGCCTGCATCTATTTCTCCTTTAGCCTAACG
	Reverse Flanking	phi3T_97-3F	GCGAAGCAGTGACAGGAGCCTCGTTAATCCCTTGATAGGGATTAATCTTACATAAC
		phi3T_97-4R	GCACTAATTTCAGCATCTTCTGTTG
*aim*X::*erm*	Forward Flanking	phi3T_aimX-1F	GTGAATTTGGATTAATGAAGAGCACTTC
		phi3T_aimX-2R	GTTCTCTCTCCTTTCTCGCCTGCTCTAATAACCCCCATGTTCTTATTTTTTG
	Reverse Flanking	phi3T_aimX-3F	GCGAAGCAGTGACAGGAGCCTCGGCATATTGAAACTAAATAAAATAGACATTTTAAACAC
		phi3T_93-8R	GCTGATTTGTCATCCCTATTTCTCAAATC
*yos*L::*erm*	Forward Flanking	YosL-phi3T-4m	AGAAGTTGCGGACGATGACGG
		YosL-phi3T-6c	GCGTTCTCTCTCCTTTCTCGCCTGCGCTGCCCCCATTTTACATCTCC
	Reverse Flanking	YosL-pih3T-7m	GCGAAGCAGTGACAGGAGCCTCGGGAGCCTATAACGATTAGTGATC
		YosL-SPBeta-8c	AATGCTACTCCTCCACCTAGC
*maz*F::*erm*	MazF-7m		TTCATCTGAAGGTCGATACGG
	MazF-8c		CAATGAAACGATTTTTCGATCCG
*maz*EF::*erm*	Forward Flanking	MazF-7m	TTCATCTGAAGGTCGATACGG
		MazE-4c	GTTCTCTCTCCTTTCTCGCCTGCCAAAAAACATACACCTCCACC
	Reverse Flanking	MazF-13m	GCGAAGCAGTGACAGGAGCCTCGTAGACATATTTGCAGGTTGC
		MazF-8c	CAATGAAACGATTTTTCGATCCG

Plasmids	Oligonucleotides	Sequence (5’-3’)
pDR110-*tet*L	tetL-1mE	CCGGAATTCATAGTGTACGTAAAAAGATTAAATTATTGCTTGG
	tetL-2cA	CCCGCGATCGCCTGTTATAAAAAAAGGATCAATTTTGAACTCTC
pDG1663+	MCS-3mE	CCGGAATTCGAGCGGCCGCTAAGCTTCTACTAGTGCTAGCGCATGCAGGATCC

Supplementary Table S5. Primers used in this study (continuation).

Plasmids	Oligonucleotides		Sequence (5'-3')
pDG1663+ P_{spank}	Pspank-4mE		CCGGAATTCGGTAAATGTGAGCACTCACAATTC
	Pspank-5cNotI		ATAAGAATGCGGCCGCAAGCTTAATTGTTATCCGCTC
pJP2808	AimP-phi3T-10mSpeI		GGACTAGTGTAATTCTTACGGCTTTAGC
	AimX-phi3T-8cB		CGCGGATCCTCCGCTTCATTCTCAAGATGGTTGTCCTC
pJP2809	AimP-phi3T-10mSpeI		GGACTAGTGTAATTCTTACGGCTTTAGC
	AimX-phi3T-8cB		CGCGGATCCTCCGCTTCATTCTCAAGATGGTTGTCCTC
pJP2810	AimP-phi3T-10mSpeI		GGACTAGTGTAATTCTTACGGCTTTAGC
	AimX-phi3T-8cB		CGCGGATCCTCCGCTTCATTCTCAAGATGGTTGTCCTC
pJP2811	AimP-phi3T-11mSpeI		GGACTAGTTATTAAACCTTATCTCTATTTAGGTTTG
	AimX-phi3T-8cB		CGCGGATCCTCCGCTTCATTCTCAAGATGGTTGTCCTC
pJP2812	AimP-phi3T-24mSpeI		GGACTAGTGTTTGACAAATTTGAAAGGAGGTGAGAATATTG
	AimP-phi3T-23cB		CGCGGATCCCCTCTTGAGCAATTATTGAATTGATTGCAGCC
pJP2813	PCR A	AimP-phi3T-11mSpeI	GGACTAGTTATTAAACCTTATCTCTATTTAGGTTTG
		AimX-phi3T-5c	TTTTGCCATTTCTTCATAAGATATTGCTTTACCTAATGCTCTTTTCATTCTAATAACCCCCttttttTTATTTTTTGATTTTTGAATTTCTGttttttTCCCTTAAAATTAGAAAAGACG
	PCR B (template PCR A)	AimP-phi3T-11mSpeI	GGACTAGTTATTAAACCTTATCTCTATTTAGGTTTG
		AimX-phi3T-9cB	CGCGGATCCTCCGCTTCATTCTCAAGATGGTTGTCCTCTTGAGCAATTATTGAATTGATTGCAGCCATTTCCTCGTACCCTTTTGCCATTTCTTCATAAGATATTGCTTTACCTAATGCT
pJP2814	PCR A	AimP-phi3T-11mSpeI	GGACTAGTTATTAAACCTTATCTCTATTTAGGTTTG
		AimP-phi3T-18c	GATTTTTGAATTTCTGGAACTTTCCCTtcatttatcatcatcatctttataatcgatatcgtgatctttataatcgccatcgtgatctttataatcTAAAATTAGAAAAGACGCCTGATC
	PCR B	AimP-phi3T-14m	GATCAGGCGTCTTTTCTAATTTTAgattataaagatcacgatggcgattataaagatcacgatatcgattataaagatgatgatgataaatgaAGGGAAAGTTCCAGAAATTCAAAAATC
		AimX-phi3T-8cB	CGCGGATCCTCCGCTTCATTCTCAAGATGGTTGTCCTC
	PCR C (template PCR A + PCR B)	AimP-phi3T-11mSpeI	GGACTAGTTATTAAACCTTATCTCTATTTAGGTTTG
		AimX-phi3T-8cB	CGCGGATCCTCCGCTTCATTCTCAAGATGGTTGTCCTC

Supplementary Table S5. Primers used in this study (continuation)

Plasmids	Oligonucleotides		Sequence (5'-3')
pJP2815	PCR A	AimP-phi3T-11mSpeI	GGACTAGTTATTAAACCTTATCTCTATTTAGGTTTG
		AimP-phi3T-17c	CCCATGTTCTTATTTTTTGATTTTTGAATTTCTGGAACTTAATTAGAA<br>AAGACGCCTGATCCAATGGATCAAACGTCTATGATAGTATTATG
	PCR B	AimP-phi3T-12m	CATAATACTATCATAGACGTTTGATCCATTGGATCAGGCGTCTTTTC<br>TAATTAAGTTCCAGAAATTCAAAAATCAAAAAATAAGAACATGGG
		AimX-phi3T-8cB	CGCGGATCCTCCGCTTCATTCTCAAGATGGTTGTCCTC
	PCR C (template PCR A + PCR B)	AimP-phi3T-11mSpeI	GGACTAGTTATTAAACCTTATCTCTATTTAGGTTTG
		AimX-phi3T-8cB	CGCGGATCCTCCGCTTCATTCTCAAGATGGTTGTCCTC
pJP2816	S785-SPBeta-3mSpeI		GGACTAGTGCCACGTGGAGCATAAAATCC
	S785-SPBeta-8cB		CGCGGATCCTTAATTAGGGTGTATGTATTTGAAGTTGATC
pJP2817	S785-SPBeta-3mSpeI		GGACTAGTGCCACGTGGAGCATAAAATCC
	S785-SPBeta-8cB		CGCGGATCCTTAATTAGGGTGTATGTATTTGAAGTTGATC
pJP2821	PCR A	AimP-phi3T-11mSpeI	GGACTAGTTATTAAACCTTATCTCTATTTAGGTTTG
		AimP-phi3T-19c	GCTCTTTTCATTCTAATAACCCCCATCACTAGATGTTATTAAAACCT<br>AATATTTAAGTGATTCCCTTAAAATTAGAAAAGACGCCTGATCC
	PCR B	AimP-phi3T-15m	GGATCAGGCGTCTTTTCTAATTTTAAGGGAATCACTTAAATATTAGG<br>TTTTAATAACATCTAGTGATGGGGGTTATTAGAATGAAAAGAGC
		AimX-phi3T-8cB	CGCGGATCCTCCGCTTCATTCTCAAGATGGTTGTCCTC
	PCR C (template PCR A + PCR B)	AimP-phi3T-11mSpeI	GGACTAGTTATTAAACCTTATCTCTATTTAGGTTTG
		AimX-phi3T-8cB	CGCGGATCCTCCGCTTCATTCTCAAGATGGTTGTCCTC
pJP2822	phi3T_aimX-7F-SalI		ACGCGTCGACTTTTAAGGGAAAGTTCCAGAAATTC
	phi3T_aimX-8R-SphI		ACATGCATGCTCCGTTGCCAATAGATTATGC
pJP2823	PCR A	phi3T_aimX-12F	gGAaGAtAAtCActtgGAaAAcGAgGCaGAgATGATcAAgACgcggTAcAAg<br>ACattaGCgagtTGAGCATATTGAAACTAAATAAAATAGACATTTTAAAC<br>ACATCG
		phi3T_aimX-8R-SphI	ACATGCATGCTCCGTTGCCAATAGATTATGC
	PCR B (template PCR A)	phi3T_aimX-11F	CgcTgGGgAAgGCgATcagcTAcGAgGAgATGGCcAAgGGtTAtGAaGAg<br>ATGGCgGCcATaAAcagtATcATaGCgCAgGAaGAtAAtCActtgGAaAAcG<br>AgGCaGAgATGATcAAgA
		phi3T_aimX-8R-SphI	ACATGCATGCTCCGTTGCCAATAGATTATGC
	PCR C (template PCR B)	phi3T_aimX-10F-SalI	ACGCGTCGACTTTTAAGGGAAAGTTCCAGAAATTCAAAAATCAAAA<br>AATAAGAACATGGGGGTTATTAGAATGAAgAGgGCgcTgGGgAAgGC<br>gATcagcTAcGAgGAgATGGCcAAgGG
		phi3T_aimX-8R-SphI	ACATGCATGCTCCGTTGCCAATAGATTATGC
pJP2824	phi3T_aimX-9F-SalI		ACGCGTCGACTTTTAAGGGAAAGTTCCAGAAATTCAAAAATCAAAA<br>AATAAGAACATGGGGGTTATTAGAATGAAAAGAGCATAATGATAAG<br>CAATATCTTATGAAGAAATGGCAAAAGG
	phi3T_aimX-8R-SphI		ACATGCATGCTCCGTTGCCAATAGATTATGC

Supplementary Table S5. Primers used in this study (continuation)

Plasmids	Oligonucleotides		Sequence (5'-3')
pJP2825	PCR A	phi3T_aimX-14F	GCATTAGGTAAAGCAATATCTTATGAAGAAgacGCAAAAGGGTACGAGGAAATGGCTGCAATC
		phi3T_aimX-8R-SphI	ACATGCATGCTCCGTTGCCAATAGATTATGC
	PCR B (template PCR A)	phi3T_aimX-13F-SalI	ACGCGTCGACTTTTAAGGGAAAGTTCCAGAAATTCAAAAATCAAAAAATAAGAACATGGGGGTTATTAGAATGAAAAGAGCATTAGGTAAAGCAATATC
		phi3T_aimX-8R-SphI	ACATGCATGCTCCGTTGCCAATAGATTATGC
pJP3004	YosL-phi3T-3mS		ACGCGTCGACCAGAGATAGAAGCTGCGCAAGC
	YosL-SPBeta-6cSphI		ACATGCATGCGTTTTACTCTCATATGTAATGATCAC
pJP3005	MazF-19mS		ACGCGTCGACAGGAGGATAATCATTTGATTGTGAAACGC
	MazF-12SphI		ACATGCATGCGCAACCTGCAAATATGTCTAAAAATC
pJP3006	PCR A	MazF-19mS	ACGCGTCGACAGGAGGATAATCATTTGATTGTGAAACGC
		MazF-10c	GGTAATTTCGCTTTCTGTATTTGTGCTGCTATGGCTGCAACAATAGCAGTTGG
	PCR B	MazF-11m	CCAACTGCTATTGTTGCAGCCATAGCAGCACAAATACAGAAAGCGAAATTACC
		MazF-12SphI	ACATGCATGCGCAACCTGCAAATATGTCTAAAAATC
pJP2826	phi3T_97-8F		ACGCGTCGACCAATTAAATGCTGCTATGAAGAAAAAACTG
	phi3T_97-9R		ACATGCATGCCACGATTCAGCTCATATTTATGTTGG
pJP2965	phi3T_97-8F		ACGCGTCGACCAATTAAATGCTGCTATGAAGAAAAAACTG
	phi3T_97-9R		ACATGCATGCCACGATTCAGCTCATATTTATGTTGG
pJP2827	phi3T_93-9F		ACGCGTCGACGAGAAGAAATCATAAAGAGCATATGC
	phi3T_93-10R		ACATGCATGCCTTCATTAAGTCATAACCGTCTAGC
pJP2829	PCR A	phi3T_93-9F	ACGCGTCGACGAGAAGAAATCATAAAGAGCATATGC
		phi3T_93-17R	CCTGGTTGCTCTAAAAATTTATCGACAATCATATGCTTTTTCCAGAC
	PCR B (template PCR A)	phi3T_93-18F	GTCTGGAAAAAGCATATGATTGTCGATAAATTTTTAGAGCAACCAGG
		phi3T_93-10R	ACATGCATGCCTTCATTAAGTCATAACCGTCTAGC
	PCR C (template PCR B)	phi3T_93-9F	ACGCGTCGACGAGAAGAAATCATAAAGAGCATATGC
		phi3T_93-10R	ACATGCATGCCTTCATTAAGTCATAACCGTCTAGC

Supplementary Table S5. Primers used in this study (continuation)

Plasmids	Oligonucleotides	Sequence (5'-3')
pLIC-AimX	AimX_FW_pLIC	AGAACCTGTACTTCCAATCCATGAAAAGAGCATTAGGTAAAGC
	AimX_Rv_pLIC	ATCCGTATCCACCTTTACTTTCATGAAGCCAGGGTTTTATAC
pLIC-AimX	AimX_FW_pLIC	AGAACCTGTACTTCCAATCCATGAAAAGAGCATTAGGTAAAGC
	AimX_Rv_pLIC	ATCCGTATCCACCTTTACTTTCATGAAGCCAGGGTTTTATAC
pLIC-phi3T-97	phi3T_97_FW	AGAACCTGTACTTCCAATCCATGAGTGAACTATACAATGCTG
	phi3T_97_RV	ATCCGTATCCACCTTTACTTTTAATCAGCAATCTTTTCTGATTG
pLIC-phi3T_93	phi3T_93_Fw	AGAACCTGTACTTCCAATCCATGACTAACAATAAATATTATACTGAGGAAAA
	phi3T_93_RV	ATCCGTATCCACCTTTACTTTCAATGAACAGCTGAGTATTGTTT
pLIC-phi3T_93L23D	phi3T_93 L23D_FW	TATGATTGTCGATAAATTTTTAGAGCAACCAG
	phi3T_93 _L23D_RV	TGCTTTTTCCAGACTTTC
pLIC-YosLSPB	YosLSPB-Plic_FW	TACTTCCAATCCATGGGGGCAGCTAGAC
	YosLSPB-Plic_RV	TATCCACCTTTACTGCTAATCGTTATAAGCCCCTGT
pLIC-YosLPhi3T	YosLPhi3T-Plic_FW	ATGGGGGCAGCTAGACG
	YosLPhi3T-Plic_RV	CTAATCGTTATAGGCTCCTGTAATTAATCTTG
pLIC-MazF	MazF_FW	AGAACCTGTACTTCCAATCCATGATTGTGAAACGCGGCGATG
	MazF_RV	ATCCGTATCCACCTTTACTTCTAAAAATCAATGAGCCAAACTG
pLIC-MazFT48A	MazFT48A_FW	TGCAGCCATAGCAGCACAAAT
	MazFT48A_RV	ACAATAGCAGTTGGGCTG
pLIC-MazE	MazE_FW	AGAACCTGTACTTCCAATCCATGTCTGAATCCAGCGCAAGAA
	MazE_RV	ATCCGTATCCACCTTTACTTTTATCCTCCGCTGACTAAGCGC
-	AimP-phi3T-40m	TGGTTTAGTAATTCTTACGGC
-	AimX-phi3T-GSP2	GCCATTTCCTCGTACCCTTTTGC
-	gyrB_B. sub-F	CGGTCGTAAACGCACTATC
-	gyrB_B. sub-R	AGGGTCCGGGACAAAATGTGT

Supplementary Table S6. Commercial reagents used in this study.

Reagent	Supplier	Catalog number	CAS number
LB Miller	Sigma-Aldrich	L3522-1KG	N/A
LB Lennox	Sigma-Aldrich	L3022-1KG	N/A
bacteriological agar	VWR	84609.0500	9002-18-0
erythromycin	Sigma-Aldrich	E6376-25G	114-07-8
kanamycin sulfate	Sigma-Aldrich	60615-5G	70560-51-9
ampicillin sodium salt	Sigma-Aldrich	A9518-25G	69-52-3
spectinomycin dihydrochloride pentahydrate	Sigma-Aldrich	S4014-5G	22189-32-8
tetracycline	Sigma-Aldrich	T3258-25G	60-54-8
ammonium sulfate, $(NH_4)_2SO_4$	Sigma-Aldrich	A4915-500G	7783-20-2
dipotassium hydrogen phosphate, K_2HPO_4	Fisher scientific	10509263	7758-11-4
potassium dihydrogen phosphate, KH_2PO_4	Fisher scientific	10573181	7778-77-0
trisodium citrate dihydrate	Fisher scientific	10396430	6132-04-3
D(+)-Glucose Anhydrous	Fisher scientific	10141520	50-99-7
yeast extract powder	Fisher scientific	11407541	8013-01-2
casein hydrolysate	Sigma-Aldrich	22090-100G	91079-40-2
magnesium sulphate heptahydrate, $MgSO_4 \cdot 7H_2O$	VWR	25165.26	10034-99-8
D/L tryptophan	Sigma-Aldrich	T8941-25G	73-22-3
L-Methionine	Sigma-Aldrich	M9625-25G	63-68-3
calcium chloride, $CaCl_2$	VWR	E506-500ML	10035-04-8
Isopropyl-β-D-thio-galactopyranoside (IPTG)	Sigma-Aldrich	I6758-10G	367-93-1
manganese(II) chloride tetrahydrate, $MnCl_2 \cdot 4H_2O$	Fisher scientific	10096063	13446-34-9
magnesium chloride hexahydrate, $MgCl_2 \cdot 6H_2O$	VWR	25108.26	7791-18-6
sodium phosphate dibasic heptahydrate, $Na_2HPO_4 \cdot 7H_2O$	Sigma-Aldrich	S9390-100G	7782-85-6
sodium phosphate monobasic, NaH_2PO_4	Sigma-Aldrich	S0751-100G	7558-80-7
potassium chloride, KCl	VWR	J64189.36	7447-40-7
β-mercaptoethanol	Sigma-Aldrich	M6250-10ML	60-24-2
lysozyme	Sigma-Aldrich	10837059001	12650-88-3
2-Nitrophenyl β-D-galactopyranoside (ONPG)	Sigma-Aldrich	N1127-5	369-07-3
mitomycin C	Sigma-Aldrich	M0503-2MG	50-07-7
RNAprotect bacterial reagent	Qiagen	76506	N/A
TRIzol Plus RNA purification kit	Thermo Fisher Scientific	12183555	N/A
MP Biomedicals Lysing Matrix B, 2 ml tubes	Fisher scientific	116911050	N/A
RQ1 RNase-free DNase	Promega	M6101	N/A
RNase-free DNase kit Qiagen	Qiagen	79254	N/A

High-Capacity cDNA Reverse Transcription kit	Applied Biosystems	4368814	N/A
QIAquick PCR purification kit	Qiagen	28106	N/A
Platinum Taq DNA polymerase HiFi	Thermo Fisher Scientific	11304029	N/A
sodium chloride, NaCl	VWR	27810.295	7647-14-5
Tris base	Fisher scientific	10376743	77-86-1
phosphate buffered saline, PBS tablets	Fisher scientific	10388739	7447-40-7
NEBuilder HiFi DNA Assembly master mix	New England Biolabs	E2621L	N/A
chloramphenicol	Sigma-Aldrich	C0378-100G	56-75-7
HiTrap TALON Crude 1mL column	Cytiva	28953766	N/A
Imidazole	Sigma-Aldrich	I2399-500G	288-32-4
EDTA, disodium salt	Fisher scientific	15508224	6381-92-6
3-kDa cut-off Amicon Ultra system	Sigma-Aldrich	UFC900324	N/A
PEG 8000	Sigma-Aldrich	89510-1KG	25322-68-3
Glycerol	Fisher scientific	BP229-4	56-81-5

References

1. Burkholder, P. R. & Giles, N. H. Induced biochemical mutations in Bacillus subtilis. *Am J Bot* **34**, 345–8 (1947).

2. Westers, H. *et al.* Genome engineering reveals large dispensable regions in Bacillus subtilis. *Mol Biol Evol* **20**, 2076–90 (2003).

3. Brady, A. *et al.* The arbitrium system controls prophage induction. *Current Biology* **31**, (2021).

4. Koo, B.-M. *et al.* Construction and Analysis of Two Genome-Scale Deletion Libraries for Bacillus subtilis. *Cell Syst* **4**, 291-305.e7 (2017).

5. Carniol, K., Ben-Yehuda, S., King, N. & Losick, R. Genetic dissection of the sporulation protein SpoIIE and its role in asymmetric division in Bacillus subtilis. *J Bacteriol* **187**, 3511–20 (2005).

6. Guérout-Fleury, A. M., Shazand, K., Frandsen, N. & Stragier, P. Antibiotic-resistance cassettes for Bacillus subtilis. *Gene* **167**, 335–6 (1995).

7. Guérout-Fleury, A. M., Frandsen, N. & Stragier, P. Plasmids for ectopic integration in Bacillus subtilis. *Gene* **180**, 57–61 (1996).
