# Supplementary material for: Antagonistic interactions between phage and host factors control arbitrium lysis–lysogeny decision
Source: Nat Microbiol. 2024 Jan 4;9(1):161–72. doi: 10.1038/s41564-023-01550-4 (PMC10769878; doi:10.1038/s41564-023-01550-4)
Supplement: Supplementary file 2 — Reporting Summary [file 41564_2023_1550_MOESM2_ESM.pdf]

nature portfolio

Corresponding author(s): Alberto Marina & Jose R. Penades

Last updated by author(s): Oct 15, 2023

Reporting Summary

Nature Portfolio wishes to improve the reproducibility of the work that we publish. This form provides structure for consistency and transparency in reporting. For further information on Nature Portfolio policies, see our Editorial Policies and the Editorial Policy Checklist.

Statistics

For all statistical analyses, confirm that the following items are present in the figure legend, table legend, main text, or Methods section.

n/a	Confirmed	
☐	☒	The exact sample size (*n*) for each experimental group/condition, given as a discrete number and unit of measurement
☐	☒	A statement on whether measurements were taken from distinct samples or whether the same sample was measured repeatedly
☐	☒	The statistical test(s) used AND whether they are one- or two-sided<br>*Only common tests should be described solely by name; describe more complex techniques in the Methods section.*
☐	☒	A description of all covariates tested
☐	☒	A description of any assumptions or corrections, such as tests of normality and adjustment for multiple comparisons
☐	☒	A full description of the statistical parameters including central tendency (e.g. means) or other basic estimates (e.g. regression coefficient) AND variation (e.g. standard deviation) or associated estimates of uncertainty (e.g. confidence intervals)
☐	☒	For null hypothesis testing, the test statistic (e.g. *F*, *t*, *r*) with confidence intervals, effect sizes, degrees of freedom and *P* value noted<br>*Give P values as exact values whenever suitable.*
☒	☐	For Bayesian analysis, information on the choice of priors and Markov chain Monte Carlo settings
☒	☐	For hierarchical and complex designs, identification of the appropriate level for tests and full reporting of outcomes
☒	☐	Estimates of effect sizes (e.g. Cohen's *d*, Pearson's *r*), indicating how they were calculated

Our web collection on statistics for biologists contains articles on many of the points above.

Software and code

Policy information about availability of computer code

Data collection	Synchroton Alba (Cerdanyola del Vallès, Spain), Diamond Light source (Didcot, UK) and ESRF (Grenoble, France) data collection software provided by the beamline
Data analysis	Size exclussion chromatogram figures and Statistical Analyses were carried out with the scientific software GraphPad Prism 9. Open source software (https://www.graphpad.com/scientific-software/prism/)<br><br>Crystallographic processing was carried out with programs from CCP4 (http://www.ccp4.ac.uk/), CCP4i2 (http://ccp4i2.fg.oisin.rc-harwell.ac.uk) and Phenix (https://www.phenix-online.org/), free and open source suites .<br><br>Model bilding was carried out with Coot 0.9.8.8 (https://www2.mrc-lmb.cam.ac.uk/personal/pemsley/coot/).<br><br>Size Exclusion Chromatography with Multi-Angle Light Scattering (SEC-MALS) data acquistion and analysis was carried out with Astra 7.1.2 software (https://www.wyatt.com/products/software/astra.html) from Wyatt.<br><br>Structure prediction was carried out with AlphaFold2 run online in Google ColabFold v1.5.2 (https://colab.research.google.com/github/sokrypton/ColabFold/blob/main/beta/AlphaFold2_advanced.ipynb#scrollTo=pc5-mbsX9PZC).<br><br>Proteins with structural similarity were indetified with DALI server (http://ekhidna2.biocenter.helsinki.fi/dali/).<br><br>Protein sequence homology search was carried out with BLASTp suite at the NCBI BLAST homepage (https://blast.ncbi.nlm.nih.gov).

Biolayer Interferometry (BLI) data acquistion and analysis was carried out with BLItz Pro 1.2 software (www.fortebio.com) from Fortebio

Secondary structure determination and fold recognition from protein circular dichroism spectra was carried out with BestSel (https://bestsel.elte.hu/index.php)

Figures for three-dimensional structures were generated with Pymol 2.1 (https://pymol.org/2/) or Chimera v1.15 (https://www.cgl.ucsf.edu/chimera/).

Some figures in this manuscript have been created with Biorender (Biorender.com)

All this information has been included in the manuscript.

For manuscripts utilizing custom algorithms or software that are central to the research but not yet described in published literature, software must be made available to editors and reviewers. We strongly encourage code deposition in a community repository (e.g. GitHub). See the Nature Portfolio guidelines for submitting code & software for further information.

Data

Policy information about availability of data

All manuscripts must include a data availability statement. This statement should provide the following information, where applicable:

- Accession codes, unique identifiers, or web links for publicly available datasets
- A description of any restrictions on data availability
- For clinical datasets or third party data, please ensure that the statement adheres to our policy

The atomic coordinates of the phi3T_93, His-phi3T_93, phi3T_93L23D and AimX-phi3T_93 complex has been deposited with PDB codes 8ANT, 8ANU, 8C8E and 8ANV, respectively, at http://www.pdb.org. The previously determined structures used in this study are available from the PDB (http://www.pdb.org) under the accession codes indicated. The rest of the data are available in the main text, supplementary materials and auxiliary files. Plasmids and bacterial strains generated during this work are listed in Supplementary Tables S3 and S4 and are available upon request. Correspondence and requests for materials can be addressed to A.M. (amarina@ibv.csic.es) or J.R.P. (j.penades@imperial.ac.uk). Source data are provided with this paper.

Research involving human participants, their data, or biological material

Policy information about studies with human participants or human data. See also policy information about sex, gender (identity/presentation), and sexual orientation and race, ethnicity and racism.

Reporting on sex and gender	N/A
Reporting on race, ethnicity, or other socially relevant groupings	N/A
Population characteristics	N/A
Recruitment	N/A
Ethics oversight	N/A

Note that full information on the approval of the study protocol must also be provided in the manuscript.

Field-specific reporting

Please select the one below that is the best fit for your research. If you are not sure, read the appropriate sections before making your selection.

☒ Life sciences ☐ Behavioural & social sciences ☐ Ecological, evolutionary & environmental sciences

For a reference copy of the document with all sections, see nature.com/documents/nr-reporting-summary-flat.pdf

Life sciences study design

All studies must disclose on these points even when the disclosure is negative.

Sample size	No calculations for determining sample size were required for this study. Experiments were repeated at least 3-times within defined comparison groups (e.g. wild type against mutant) and followed by statistical analyses described.
Data exclusions	No data was excluded from the analyses.
Replication	At least 3 independent experiments were carried out in the bacteriophage induction, infection, tittering and lysogenisation experiments as well as reporter assays and growth experiments, as indicated in each figure description. Experimental observations were reproducible for all replication attempts.<br>The RT-PCR experiment was performed only 1 time, as indicated in figure description.

	Binding affinities were measured by BLI three times for each sample. No discrepancies superior of 10 % were observed between samples. SEC-MALS assays were carried two times for each sample. No discrepancies superior of 5 % were observed between samples.
Randomization	Not relevant for our study. Samples not allocated in sample groups. Independent bacterial cultures for each bacterial strain were cultured.
Blinding	The results reported in this study are not related to a clincal research study, thus blinding was not necessary

Reporting for specific materials, systems and methods

We require information from authors about some types of materials, experimental systems and methods used in many studies. Here, indicate whether each material, system or method listed is relevant to your study. If you are not sure if a list item applies to your research, read the appropriate section before selecting a response.

Materials & experimental systems

n/a	Involved in the study
☒	☐ Antibodies
☒	☐ Eukaryotic cell lines
☒	☐ Palaeontology and archaeology
☒	☐ Animals and other organisms
☒	☐ Clinical data
☒	☐ Dual use research of concern
☒	☐ Plants

Methods

n/a	Involved in the study
☒	☐ ChIP-seq
☒	☐ Flow cytometry
☒	☐ MRI-based neuroimaging
